# Crystal structure and RNA-binding properties of an Hfq homolog from the deep-branching Aquificae: conservation of the lateral RNA-binding mode

**DOI:** 10.1107/S2059798317000031

**Published:** 2017-03-31

**Authors:** Kimberly A. Stanek, Jennifer Patterson-West, Peter S. Randolph, Cameron Mura

**Affiliations:** a Department of Chemistry, University of Virginia, 409 McCormick Road, Charlottesville, VA 22904, USA

**Keywords:** Hfq, Sm protein, RNA, *Aquifex aeolicus*, hexamer, evolution

## Abstract

The host factor Hfq, as the bacterial branch of the Sm family, is an RNA-binding protein involved in the post-transcriptional regulation of mRNA expression and turnover. Hfq facilitates pairing between small regulatory RNAs (sRNAs) and their corresponding mRNA targets by binding both RNAs and bringing them into close proximity. Hfq homologs self-assemble into homo-hexameric rings with at least two distinct surfaces that bind RNA. Recently, another binding site, dubbed the ‘lateral rim’, has been implicated in sRNA·mRNA annealing; the RNA-binding properties of this site appear to be rather subtle, and its degree of evolutionary conservation is unknown. An Hfq homolog has been identified in the phylogenetically deep-branching thermophile *Aquifex aeolicus* (*Aae*), but little is known about the structure and function of Hfq from basal bacterial lineages such as the Aquificae. Therefore, *Aae* Hfq was cloned, overexpressed, purified, crystallized and biochemically characterized. Structures of *Aae* Hfq were determined in space groups *P*1 and *P*6, both to 1.5 Å resolution, and nanomolar-scale binding affinities for uridine- and adenosine-rich RNAs were discovered. Co-crystallization with U_6_ RNA reveals that the outer rim of the *Aae* Hfq hexamer features a well defined binding pocket that is selective for uracil. This *Aae* Hfq structure, combined with biochemical and biophysical characterization of the homolog, reveals deep evolutionary conservation of the lateral RNA-binding mode, and lays a foundation for further studies of Hfq-associated RNA biology in ancient bacterial phyla.

## Abstract 2

The structure of an Hfq homolog from the deep-branching thermophilic bacterium *Aquifex aeolicus*, determined to 1.5 Å resolution both in the apo form and bound to a uridine-rich RNA, reveals a conserved, pre-organized RNA-binding pocket on the lateral rim of the Hfq hexamer.

## 1. Introduction   

The bacterial protein Hfq, initially identified as an *Escherichia coli* host factor required for the replication of RNA bacteriophage Qβ (Franze de Fernandez *et al.*, 1968 ▸, 1972 ▸), is now known to play a central role in the post-transcriptional regulation of gene expression and mRNA metabolism (Vogel & Luisi, 2011 ▸; Sauer, 2013 ▸; Updegrove *et al.*, 2016 ▸). Hfq has been linked to many RNA-regulated cellular pathways, including stress response (Sledjeski *et al.*, 2001 ▸; Zhang *et al.*, 2002 ▸; Fantappie *et al.*, 2009 ▸), quorum sensing (Lenz *et al.*, 2004 ▸) and biofilm formation (Mandin & Gottesman, 2010 ▸; Mika & Hengge, 2013 ▸). The diverse cellular functions of Hfq stem from its fairly generic role in binding small, noncoding RNAs (sRNAs) and facilitating base-pairing interactions between these regulatory sRNAs and target mRNAs. A given sRNA might either upregulate (Soper *et al.*, 2010 ▸) or downregulate (Ikeda *et al.*, 2011 ▸) one or more target mRNAs *via* distinct mechanisms. For example, the sRNA RhyB downregulates several Fur-responsive genes under iron-limiting conditions (Masse & Gottesman, 2002 ▸), whereas the DsrA, RprA and ArcZ sRNAs stimulate translation of *rpoS* mRNA, encoding the stationary-phase σ^s^ factor (Soper *et al.*, 2010 ▸). In general, Hfq is required for cognate sRNA·mRNA pairings to be productive, and abolishing Hfq function typically yields pleiotropic phenotypes, including diminished viability (Fantappie *et al.*, 2009 ▸; Vogel & Luisi, 2011 ▸).

**Figure 1:**
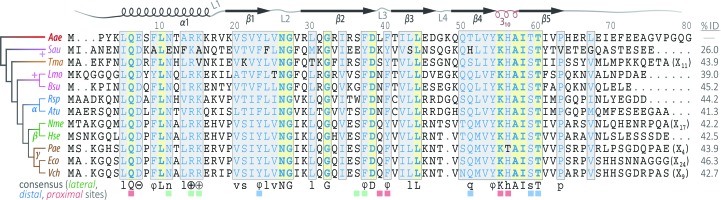
Multiple sequence alignment of *Aae* Hfq and some representative homologs. Sequence analysis of several Hfq homologs, characterized from various phyla, reveals the conservation of key amino acids comprising the three distinct RNA-binding regions of Hfq (distal, proximal and lateral). The *Aae* Hfq sequence is numbered at the top, and secondary-structural elements are drawn based on the *Aae* Hfq crystal structures reported here; helices are schematized as spirals, strands as arrows and numbered loop labels are shown (a short 3_10_-helix forms loop L5, colored brown). Strictly identical amino acids are in bold blue text on a yellow background, while sites with highly similar residues are highlighted with a gray background; these blocks of partially conserved residues are also lightly boxed. In the consensus sequence shown at the bottom, uppercase letters indicate strict identity and lowercase letters correspond to physicochemically equivalent residues that meet a similarity threshold (≥85% of sites in a given column). Residues known to contact RNA at the proximal, distal or lateral sites are marked with red, blue or green square symbols, respectively. Note the high level of conservation of residues involved in all three RNA-binding sites. In addition to *Aae* Hfq (from the phylum Aquificae), the 12 aligned sequences include (i) three Hfq homologs from the mostly Gram-positive Firmicutes (*Sau*, *Lmo* and *Bsu*), (ii) a homolog from the ancient phylum Thermotogae and (iii) several characterized Hfq orthologs from the α-, β- and γ-proteobacteria. The relationships between these species are indicated in the dendrogram (left) obtained during the progressive alignment calculation and colored so as to highlight phylum-level differences. The genus/species and sequence accession codes (GenBank) are as follows: *Aae*, *A. aeolicus* (AAC06479.1); *Sau*, *Staphylococcus aureus* (ADC37472.1); *Tma*, *T. maritima* (AGL49448.1); *Lmo*, *Listeria monocytogenes* (CBY70202.1); *Bsu*, *Bacillus subtilis* (BAM57957.1); *Rsp*, *Rhodobacter sphaeroides* (A3PJP5.1); *Atu*, *Agrobacterium tumefaciens* (EHH08904.1); *Nme*, *Neisseria meningitidis* (P64344.1); *Hse*, *Herbaspirillum seropedicae* (ADJ64436.1); *Pae*, *Pseudomonas aeruginosa* (B3EWP0.1); *Eco*, *Escherichia coli* (BAE78173.1); *Vch*, *Vibrio cholerae* (A5F3L7.1).

Hfq is the bacterial branch of the Sm superfamily of RNA-associated proteins (Mura *et al.*, 2013 ▸). Eukaryotic Sm and Sm-like (LSm) proteins act in intron splicing and other mRNA-related processing pathways (Will & Luhrmann, 2011 ▸; Tharun, 2009 ▸; Tycowski *et al.*, 2006 ▸), while the cellular functions of Sm homologs in the archaea remain unclear. Although the biological functions and amino-acid sequences of Sm proteins vary greatly, the overall Sm fold is conserved across all three domains of life: five antiparallel β-strands form a highly bent β-sheet, often preceded by an N-terminal α-helix (Fig. 1 ▸; Kambach *et al.*, 1999 ▸). Sm proteins typically form cyclic oligomers *via* hydrogen bonding between the β4 and β5′ (edge) strands of monomers in a head-to-tail manner, yielding a toroidal assembly of six (Hfq) or seven (other Sm) subunits (Mura *et al.*, 2013 ▸); Hfq and other Sm rings can further associate into head-to-head and head-to-tail stacked rings, as well as polymeric assemblies (Arluison *et al.*, 2006 ▸). The oligomerization mechanism also varies across the Sm superfamily: Sm-like archaeal proteins (SmAPs) and Hfq homologs spontaneously self-assemble into stable homo-heptameric or homo-hexameric rings (respectively) that resist chemical and thermal denaturation, whereas eukaryotic Sm hetero-heptamers form *via* a chaperoned biogenesis pathway. This intricate assembly pathway (Fischer *et al.*, 2011 ▸) involves staged interactions with single-stranded RNA (*e.g.* small nuclear RNAs of the spliceosomal snRNPs), such that RNA threads through the central pore of the Sm ring (Leung *et al.*, 2011 ▸). In contrast, Hfq hexamers expose two distinct RNA-binding surfaces (Mikulecky *et al.*, 2004 ▸), termed the ‘proximal’ and ‘distal’ (with respect to the α-helix) faces of the ring. These two surfaces can bind RNA independently and simultaneously (Wang *et al.*, 2013 ▸), with different RNA sequence specificities along each face.

The proximal face of Hfq preferentially binds uridine-rich single-stranded RNA (ssRNA) in a manner that is well conserved amongst Gram-positive bacteria (Schumacher *et al.*, 2002 ▸; Kovach *et al.*, 2014 ▸) and Gram-negative bacteria (Weichenrieder, 2014 ▸). The binding region, located near the pore, consists of six equivalent ribonucleotide-binding pockets, and can thus accommodate a six-nucleotide segment of ssRNA. Each uracil base π-stacks with a conserved aromatic side chain (Phe or Tyr) from the L3 loops of adjacent monomers (*e.g.* Phe42 in *E. coli*, corresponding to Phe40 in *Aquifex aeolicus*; Fig. 1 ▸), and nucleobase specificity is achieved *via* hydrogen bonding between Gln8 and the exocyclic O2 of each uracil. (Unless otherwise noted, residue numbers refer to the *E. coli* Hfq sequence; for clarity, only the *Aae* numbering is shown in Fig. 1 ▸.) A key physiological function of the proximal face of Hfq is thought to be the selective binding of the U-rich 3′-termini of sRNAs, resulting from rho-independent transcription termination (Wilson & von Hippel, 1995 ▸). The recognition of these 3′ ends by Hfq is facilitated by the well conserved His57 of the L5 loop (‘3_10_-helix’ in Fig. 1 ▸), which is well positioned to interact with the unconstrained, terminal 3′-hydroxyl group (Sauer & Weichenrieder, 2011 ▸; Schulz & Barabas, 2014 ▸). This mode of recognition may also explain the ability of Hfq to bind specifically to sRNAs over DNA or other RNAs.

In contrast to the uracil-binding proximal region, the distal face of Hfq preferentially binds adenine-rich RNA, with the mode of binding varying between Gram-negative and Gram-positive species. Hfq homologs from Gram-negative bacteria specifically recognize RNAs with a trinucleotide motif, denoted (A–R–N)*_n_*, where A is adenine, R is purine and N is any nucleotide; this recognition element was recently refined to be a more restrictive (A–A–N)*_n_* motif (Robinson *et al.*, 2014 ▸). A–A–N-containing RNAs bind to a large surface region on the distal face, which can accommodate up to 18 nucleotides of an ssRNA (Link *et al.*, 2009 ▸), and such RNAs are recognized in a tripartite manner: (i) the first A-site is formed by residues between the β2 and β4 strands of one monomer (Glu33 ensures adenine specificity), (ii) the second A site lies between the β2 strands of adjacent subunits, and includes a conserved Tyr25 (Fig. 1 ▸) that engages in π-stacking inter­actions, and (iii) a nonspecific (N) nucleotide binding site bridges to the next A–A pocket. In contrast to this recognition mechanism, the distal face of Gram-positive Hfq recognizes a bipartite adenine-linker (AL)*_n_* motif. This structural motif features an A-site that is similar to the second A-site of Gram-negative bacteria; in addition, a nonspecific nucleotide-binding pocket acts as a linker (L) site, allowing 12 nucleotides to bind in a circular fashion atop this face of the hexamer (Horstmann *et al.*, 2012 ▸; Someya *et al.*, 2012 ▸). The ability of the distal face to specifically bind A-rich regions, such as the long, polyadenylated 3′-tails of mRNAs (Folichon *et al.*, 2003 ▸), leads to several links between Hfq and mRNA degradation/turnover pathways (Mohanty *et al.*, 2004 ▸; Bandyra & Luisi, 2013 ▸; Régnier & Hajnsdorf, 2013 ▸). The general capacity of Hfq to independently bind RNAs at the proximal and distal sites brings these distinct RNA species into close proximity as part of an sRNA·Hfq·mRNA ternary complex. Indeed, a chief cellular role of Hfq is the productive annealing of RNA strands in this manner, for whatever downstream physiological purpose (be it stimulatory or inhibitory).

Independent binding of RNAs at the proximal/distal sites elucidates only part of what is known about the RNA-related activities of Hfq. For instance, Hfq has been shown to protect internal regions of sRNA (Balbontín *et al.*, 2010 ▸; Ishikawa *et al.*, 2012 ▸; Updegrove & Wartell, 2011 ▸; Zhang *et al.*, 2002 ▸) and to reduce the thermodynamic stability (Δ*G* ^o^_fold_) of some RNA hairpins (Robinson *et al.*, 2014 ▸), but current mechanistic models of Hfq activity do not account for all of these properties. In addition, recent studies have identified a new RNA-binding site on the Hfq ring beyond the proximal and distal sites (Sauer, 2013 ▸). This third site, located on the outer rim of the Hfq toroid and presaged in RNA-binding studies a decade ago (Sun & Wartell, 2006 ▸), is variously termed the ‘lateral’, ‘rim’ or ‘lateral rim’ site (the terms are used synonymously herein). Mutational analyses reveal that an arginine-rich patch near the N-terminal α-helix, containing the segment R^16^R^17^E^18^R^19^ in *E. coli*, facilitates rapid annealing of Hfq-bound mRNAs and sRNAs (Panja *et al.*, 2013 ▸). These arginine residues, along with conserved aromatic (Phe/Tyr39; ‘φ’ in Fig. 1 ▸) and basic (Lys47) residues, look to be vital for the binding of full-length sRNAs to Hfq (Sauer *et al.*, 2012 ▸). Further understanding of the precise mechanism of RNA binding to the lateral rim site (and any base specificity at this site) has been hindered by a lack of structural information on Hfq_rim_⋯RNA interactions. A recent crystal structure of *E. coli* Hfq complexed with the full-length riboregulatory sRNA RydC (a regulator of biofilms and some mRNAs) revealed a potential binding pocket formed by Asn13, Arg16, Arg17 and Phe39, and capable of accommodating two nucleotides of uridine (Dimastrogiovanni *et al.*, 2014 ▸); however, the exact positioning and geometry of the nucleotides were not discernible at the resolution (3.5 Å) of this model.

Our current mechanistic knowledge of Hfq⋯RNA inter­actions is based primarily on homologs from proteobacterial species, particularly the γ-proteobacteria *E. coli* and *Pseudomonas aeruginosa*; structural information about nucleotide binding at the lateral site is available only from these two species. We do not know whether the rim RNA-binding mode is conserved in homologs from other bacterial species, or perhaps even more broadly (in archaeal and eukaryotic lineages). Hfq orthologs from phylogenetically deep-branching bacteria, such as *Aae*, may help clarify the degree of conservation of the various RNA-binding surfaces of Hfq, including the lateral rim. *Aae* Hfq has been shown, *via* immunoprecipitation/deep-sequencing studies, to partially restore the phenotype of a *Salmonella enterica* Hfq knockout strain, Δ*hfq* (Sittka *et al.*, 2009 ▸), but nothing else is known about the RNA-binding properties of *Aae* Hfq. Precisely positioning *Aae* within the bacterial phylogeny is difficult given, for instance, that many *Aae* genes are similar to those in ∊-proteobacteria (Eveleigh *et al.*, 2013 ▸). Nevertheless, 16S rRNA and genomic sequencing data firmly place *Aae*, along with other members of the Aquificales order, among the deepest branches in the bacterial tree, near the bacterial/archaeal divergence. Sequence similarity to proteobacterial genes has been attributed to extensive lateral gene transfer (Oshima *et al.*, 2012 ▸; Boto, 2010 ▸); importantly, extensive lateral transfer does not seem to have occurred with Hfq homologs (Sun *et al.*, 2002 ▸), and Sm proteins are likely to have a single, well defined origin (Veretnik *et al.*, 2009 ▸).

Here, we report the crystal structure and RNA-binding properties of an *A. aeolicus* Hfq ortholog. *Aae* Hfq crystallized in multiple space groups, with both hexameric and dodecameric assemblies in the lattices. These oligomeric states were further examined in solution *via* chemical cross-linking assays, analytical size-exclusion chromatography and light-scattering experiments. We found that *Aae* Hfq binds uridine-rich and adenosine-rich RNAs with nanomolar affinities *in vitro*, and that the inclusion of Mg^2+^ enhances the binding affinities by factors of approximately two (A-rich) or approximately ten (U-rich). Co-crystallization of *Aae* Hfq with U_6_ RNA reveals well defined electron density (to 1.5 Å resolution) for at least two ribonucleotides in a rim site, suggesting that this auxiliary RNA-binding site is conserved even amongst evolutionarily ancient bacteria. Finally, comparative structural analysis reveals that (i) the spatial pattern of Hfq⋯RNA interatomic contacts, which effectively defines the rim site, is preserved between *Aae* and *E. coli*, and (ii) the residues comprising the *Aae* Hfq rim site are pre-organized for U-rich RNA binding.

## 2. Materials and methods   

### 2.1. Cloning, expression and purification of *Aae* Hfq   

The *Aae hfq* gene was cloned *via* the polymerase incomplete primer extension (PIPE) methodology (Klock & Lesley, 2009 ▸) using an *A. aeolicus* genomic sample as a PCR template. The T7-based expression plasmid pET-28b(+) was used, yielding a recombinant protein construct bearing an N-terminal 6×His tag and a thrombin-cleavable linker preceding the Hfq (Supplementary Fig. S1*a*, Supplementary Table S1); in all, the affinity tag and linker extend the 80-amino-acid native sequence by 20 residues, giving the full-length sequence in Supplementary Fig. S1(*a*). Plasmid amplification, and *in vivo* ligation of the vector and insert, were achieved *via* transformation of the PIPE products into chemically competent TOP10 *E. coli* cells. Recombinant *Aae* Hfq was produced by transforming the plasmid into the *E. coli* BL21(DE3) expression strain, followed by outgrowth in Luria–Bertani medium at 310 K. Finally, expression of *Aae* Hfq from the T7 *lac*-based promoter was induced by the addition of 1 m*M* isopropyl β-d-1-thiogalactopyranoside (IPTG) when the optical density measured at 600 nm (OD_600_) reached ∼0.8–1.0. The cell cultures were then incubated at 310 K with shaking (∼230 rev min^−1^) for an additional 4 h, pelleted at 15 000*g* for 5 min at 277 K and then stored at 253 K overnight.

Cell pellets were resuspended in a solubilization and lysis buffer [50 m*M* Tris pH 7.5, 750 m*M* NaCl, 0.4 m*M* PMSF, 0.01 mg ml^−1^ chicken egg-white lysozyme (Fisher)] and incubated at 310 K for 30 min. The cells were then mechanically lysed using a microfluidizer. To remove cell debris, the lysate was pelleted *via* centrifugation at 35 000*g* for 20 min at 277 K. The supernatant from this step was then incubated at 348 K for 20 min, followed by centrifugation at 35 000*g* for 20 min; this heat-cut step was performed because most Hfq homologs examined thus far have been thermostable, and because *A. aeolicus* is a hyperthermophile (with an optimum growth temperature *T*_opt_ of ∼360 K; Huber & Eder, 2006 ▸). To reduce contamination by any spurious *E. coli* nucleic acids, which have been known to co-purify with other Hfqs, the clarified supernatant from the heating step was treated with high concentrations (∼6 *M*) of guanidinium hydrochloride (GndCl). To remove any particulate matter, Gnd-treated samples were then immediately clarified by 0.2 µm syringe filtration.

Recombinant *Aae* Hfq was then purified *via* immobilized metal-affinity chromatography (IMAC) using a Ni^2+^-charged iminodiacetic acid Sepharose column with an NGC (Bio-Rad) medium-pressure liquid-chromatography system. After loading the clarified supernatant from the heat-cut and GndCl-treatment steps, the column was treated with four column volumes of wash buffer (50 m*M* Tris pH 8.5, 150 m*M* NaCl, 6 *M* GndCl, 10 m*M* imidazole). Next, *Aae* Hfq was eluted by applying a linear gradient, from 0 to 100% over ten column volumes, of elution buffer (identical to the wash buffer but with 600 m*M* imidazole). Protein-containing fractions, as assessed by the absorbance at 280 nm and chromatogram elution profiles, were then combined and, in order to remove GndCl, dialyzed against a buffer consisting of 25 m*M* Tris pH 8.0, 1 *M* arginine. Next, to prepare for the removal of the 6×His tag, the protein was then dialyzed into 50 m*M* Tris pH 8.0, 500 m*M* NaCl, 12.5 m*M* EDTA. The *Aae* Hfq sample was subjected to proteolysis with thrombin at a 1:600 Hfq:thrombin ratio (by mass) by incubating at 315 K overnight (∼16 h), followed by application to a benzamidine affinity column to remove the thrombin. To improve the sample homogeneity, *Aae* Hfq was further purified over a preparative-grade HiPrep 16/60 Sephacryl S-300 HR gel-filtration column; *Aae* Hfq eluted as a single, well defined peak. Chromatographic steps were conducted at room temperature; lengthier incubation steps, such as dialysis, were carried out at 310 or 315 K throughout the purification, as *Aae* Hfq samples were found to be relatively insoluble over a few hours at room temperature (∼295 K).

**Figure 2:**
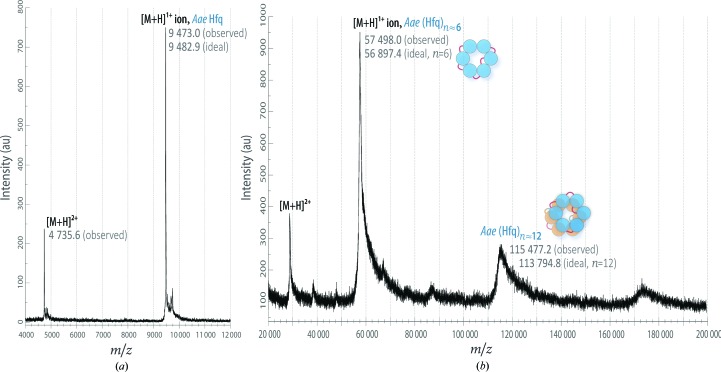
*Aae* Hfq monomers and oligomers, as assayed by cross-linking and mass spectrometry. MALDI-TOF spectra are shown for (*a*) native, untreated (non-cross-linked) *Aae* Hfq monomers, with an expected MW of 9482.9 Da based on the recombinant protein sequence (Supplementary Fig. S1), as well as (*b*) a chemically cross-linked *Aae* Hfq sample. As detailed in §[Sec sec2.2]2.2, cross-linking assays employed a gentle (‘indirect’) method, using formaldehyde as a cross-linking agent. The main peaks in the cross-linked sample correspond to hexamers and dodecamers, with expected MWs of 56 897.4 and 113 794.8 Da, respectively. The singly-charged molecular ion peaks, [*M*+H]^1+^, are accompanied by schematics (blue and orange balls) that indicate the anticipated architecture of the oligomeric states, alongside the MW of the peak as determined from the mass spectrum (cross-linked species are better characterized by a MW range, rather than a single value, because of variability in the number of cross-linker molecules that react).

*Aae* Hfq sample purity was generally assayed *via* SDS–PAGE gels or matrix-assisted laser desorption/ionization time-of-flight mass spectrometry (MALDI-TOF MS). Samples were prepared for MALDI by diluting them 1:4(*v*:*v*) with 0.01%(*v*/*v*) trifluoroacetic acid (TFA) and then spotting them onto a steel MALDI plate in a 1:1(*v*:*v*) ratio with a matrix solution (15 mg ml^−1^ sinapinic acid in 50% acetonitrile, 0.05% TFA); this mixture crystallized *in situ via* solvent evaporation. Mass spectra were acquired on a Bruker MicroFlex instrument operating in linear positive-ion mode (25 kV accelerating voltage; 50–80% grid voltage), and the final spectra were the result of averaging at least 50 laser shots. Two sets of molecular-weight calibrants were used for low (4–20 kDa) and high (20–100 kDa) *m*/*z* ranges. Purification progress and sample MALDI spectra are illustrated in Supplementary Fig. S1(*b*) and Fig. 2 ▸, respectively.

### 2.2. Cross-linking assays   

Purified *Aae* Hfq was chemically cross-linked, using formaldehyde, in a so-called ‘indirect’ (vapor-diffusion-based) method (Fadouloglou *et al.*, 2008 ▸). Firstly, *Aae* Hfq samples at 0.6 mg ml^−1^ were dialyzed into a buffer consisting of 25 m*M* HEPES pH 8.0, 500 m*M* NaCl. Reaction solutions were prepared in 24-well Linbro plates using micro-bridges (Hampton Research). Immediately before use, 5 *N* HCl was added to 25%(*w*/*v*) formaldehyde in a 1:40(*v*:*v*) ratio. Next, 40 µl of this acidified formaldehyde solution was added to the micro-bridge, and 15 µl of the 0.6 mg ml^−1^ *Aae* Hfq was added to a silanized cover slip. Greased wells were then sealed by flipping over the cover slips and the reaction was incubated at 310 K for 40 min. Reactions were quenched by the addition of a primary amine; specifically, 5 µl of 1 *M* Tris pH 8.0 was mixed into the 15 µl protein droplet. Cross-linked samples were then desalted on a C4 resin (using ZipTip pipette tips) in preparation for analysis *via* MALDI-TOF MS, as described above.

### 2.3. Analytical size-exclusion chromatography and multi-angle static light scattering   

Analytical size-exclusion chromatography (AnSEC) was performed with a pre-packed Superdex 200 Increase 10/300 GL column and a Bio-Rad NGC medium-pressure liquid-chromatography system. Prior to AnSEC, all protein samples were dialyzed into a running buffer consisting of 50 m*M* Tris pH 8.0, 200 m*M* NaCl. In separate experiments, *Aae* Hfq samples (250 µ*M* protein) were mixed in a 1:1(*v*:*v*) ratio with RNA sequences (at 50 µ*M*) denoted ‘U_6_’ [5′-monophos­phate–r(U)_6_–3′-OH] or ‘A_18_’ [5′-monophosphate–r(A)_18_–3′-­OH] and equilibrated by incubation at 310 K for 1 h prior to loading onto the AnSEC column. Elution volumes were measured by simultaneously monitoring the absorbance at 260 nm (RNA) and at 280 nm (protein). A standard curve was generated using the Sigma gel-filtration markers kit, with calibrants in the 12–200 kDa molecular-weight range: cytochrome *c* (12.4 kDa), carbonic anhydrase (29 kDa), bovine serum albumin (66 kDa), alcohol dehydrogenase (150 kDa) and β-amylase (200 kDa); blue dextran was used to calculate the void volume *V*_0_.

To determine absolute molecular masses (*i.e.* without reference standards and implicit assumptions about spheroidal shapes), and in order to assess potential polydispersity of *Aae* Hfq in solution, multi-angle static light scattering (MALS) was used in tandem with size-exclusion chromatographic (SEC) separation. A flow-cell-equipped light-scattering (LS) detector was used downstream of the SEC, inline with an absorbance detector (UV) and a differential refractive-index (RI) detector. In our SEC–UV/RI/LS system, (i) the SEC step serves to fractionate a potentially heterogeneous sample (giving the usual chromatogram, recorded at either 280 or 260 nm on a Waters UV–Vis detector), (ii) the differential refractometer (RI) estimates the solute concentration *via* changes in the solution refractive index (*i.e.* d*n*/d*c*) and (iii) the LS detector measures the excess scattered light. This workflow was executed on a Waters HPLC system equipped with the Wyatt instrumentation noted below, and utilized the same column (Superdex 200) and solution buffer conditions as described immediately above. LS measurements were taken at three detection angles using a Wyatt miniDAWN TREOS (λ = 658 nm), and the differential refractive index was recorded using a Wyatt Optilab T-rEX. This approach enables the molecular mass of the solute in each fraction to be determined because the amount of light scattered (from the LS data) scales with the weight-averaged molecular masses (the desired quantity) and solute concentrations (from the RI data); if multiple species exist in a given (heterogeneous) fraction, the polydispersity can be quantified as the ratio of the weight-averaged (*M*_w_) and number-averaged (*M*_n_) molar masses. Data were processed and analysed using the *ASTRA* software package (Wyatt), applying the Zimm formalism to extract the weight-averaged molecular masses (Folta-Stogniew, 2009 ▸).

### 2.4. Fluorescence polarization-based binding assays   

RNA-binding affinities were determined *via* fluorescence anisotropy/polarization experiments (FA/FP; Pagano *et al.*, 2011 ▸) using fluorescein-labeled oligoribonucleotides. In particular, the RNA probes 5′-FAM–r(U)_6_–3′-OH (FAM–U_6_) and 5′-FAM–r(A)_18_–3′-OH (FAM–A_18_) were used, with 6-carboxyfluorescein amidite (FAM) modification of the 5′ ends; the FAM label features absorption and emission wavelengths, λ_max_, of 485 nm (excitation) and 520 nm (detection), respectively. FAM-labeled RNAs at 5 n*M* were added to a serially diluted concentration series of purified *Aae* Hfq (in 50 m*M* Tris pH 8.0, 500 m*M* NaCl) and allowed to equilibrate for 45 min at room temperature. The highest Hfq concentration was 30 µ*M* (in terms of monomer), and a total of 18 serial dilutions were performed to produce data sets such as that in Fig. 4. For binding assays that were supplemented with Mg^2+^, a 1 *M* MgCl_2_ stock solution was used and the final Mg^2+^ concentration in the binding reaction was 10 m*M*.

The fluorescence polarization, *P*, is measured as *P* = [(*I*_∥_ − *I*_⊥_)/(*I*_∥_ + *I*_⊥_)], where *I*_∥_ and *I*_⊥_ are the emitted light intensities in directions parallel and perpendicular to the excitation plane, respectively. FP data were recorded on a PheraSTAR spectrofluorometer equipped with a plate reader (BMG Labtech), and values from three independent trials were averaged. The effective polarization, in units of millipolarization (mP), was plotted against log[(Hfq)_6_]. Binding data were fitted, *via* nonlinear least-squares regression, to a logistic functional form of the classic sigmoidal curve for saturable binding. Specifically, the four-parameter equation
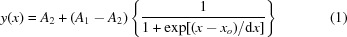
was used, where the independent variable *x* is the log of the (Hfq)_6_ concentration at a given data point and the fit parameters are (i) *A*_1_, the polarization at the end of the titration (unbound; lower plateau of the binding isotherm); (ii) *A*_2_, the final polarization at the start of the titration (saturated binding; upper plateau); (iii) *x*_0_, the apparent equilibrium dissociation constant (*K*_d,app_) for the binding reaction in terms of log[(Hfq)_6_]; and (iv) a parameter, d*x*, giving the characteristic scale/width over which the slope of the sigmoid changes. In this formulation, d*x* is essentially the classic Hill coefficient, measuring the steepness of the binding curve; the greater the magnitude of d*x*, the narrower the transition region. In addition to fitting the binding data with the four-parameter logistic model (1), a simpler, three-parameter model was also applied, with the functional form
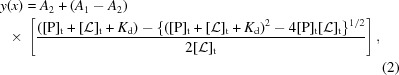
where the terms *A*_1_ and *A*_2_ are as above in (1), *K*_d_ is the dissociation constant (*x*_0_ above) and the variables 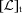 and [P]_t_ are the total concentrations of ligand (FAM-labeled RNA) and receptor (here, taken as an Hfq hexamer), respectively. Although assuming a 1:1 stoichiometry between *Aae* (Hfq)_6_ and RNA, and not capturing potential cooperativity between possibly multiple ligand-binding sites, this second model does account for the effects of receptor depletion on the fitted *K*_d_ values. This, in turn, is an important consideration in fitting data points with abscissas near (within ∼10× of) the true *K*_d_, as the assumption that the concentration of ligand·receptor complex, [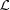
·P], is far lower than the total concentrations of each species (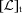, [P]_t_) is violated if [P]_t_ ≃ *K*_d_. That is, free [P] ≅ [P]_t_ no longer holds near the *K*_d_. Despite the advantage of accounting for receptor depletion, note that this treatment implicitly takes the Hill coefficient (the ‘slope factor’ for the transition region) to be 1, rather than letting it vary (as in equation 1); indeed, the only three degrees of freedom with which to describe the binding curve are the upper and lower asymptotes and the midpoint of the transition (*i.e.* *K*_d_, or ‘*x*_0_’ in equation 1). Assuming a Hill coefficient of unity and a simple (1:1 stoichiometry) 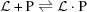 equilibrium, one can show that neglecting to account for receptor-depletion phenomena gives an apparent (fitted) dissociation constant, *K*_d,app_, that exceeds by [P]_t_/2 the ‘true’ *K*_d,app_ obtained *via* equation (2). For these reasons, both models, equations (1) and (2), were considered in fitting the data. All calculations described in this section were performed with in-house code written in the R programming language using the *RStudio* integrated development environment.

### 2.5. X-ray crystallography   

#### 2.5.1. Crystallization   

Prior to crystallization trials, purified *Aae* Hfq was dialyzed into a buffer consisting of 50 m*M* Tris pH 8.0, 500 m*M* NaCl and concentrated to 4.0 mg ml^−1^. Protein samples were typically stored at 310 K to retain solubility, and were used within two weeks of purification. All crystallization trials were performed with the vapour-diffusion method in sitting-drop format. Sparse-matrix screening (Jancarik & Kim, 1991 ▸) yielded initial leads (visible crystals) under several conditions, and these were then optimized by adjusting the concentration of protein and precipitating agent, as well as the pH of the mother liquor. Diffraction-grade crystals (Supplementary Figs. S1*c* and S1*d*) were reproducibly obtained with 0.1 *M* sodium cacodylate pH 5.5, 5%(*w*/*v*) PEG 8000, 40%(*v*/*v*) 2-methyl-2,4-pentanediol (MPD) as the crystallization buffer. In our final condition, 6 µl sitting drops (3 µl well + 3 µl of 4 mg ml^−1^ *Aae* Hfq) were equilibrated at 291 K against 600 µl wells containing the crystallization buffer. Initial microcrystals developed over several days. Optimization of the above condition *via* additive screens (Hampton Research) led to the discovery of several compounds that, in a 1:4(*v*:*v*) additive:crystallization buffer ratio, slowed nucleation and increased the crystal size. The optimized crystals grew to average dimensions of 50 × 50 × 10 µm within two weeks and adopted cubic or hexagonal plate morphologies. Three particularly useful additives, which were used in subsequent crystallization trials, were (i) 0.1 *M* hexamminecobalt(III) chloride, [Co(NH_3_)_6_]Cl_3_, (ii) 1.0 *M* GndCl and (iii) the non-ionic detergent *n*-octyl-β-d-glucoside at 5%(*w*/*v*). The final apo-form *Aae* Hfq crystals were obtained with additive (i); details are provided in Supplementary Table S2. *Aae* Hfq was also co-crystallized with a U-rich RNA (U_6_) under the above crystallization conditions and supplemented with additive (ii) instead of additive (i); these crystals were obtained by first incubating the purified protein with 500 µ*M* 5′-monophos­phate–r(U)_6_–3′-OH (hereafter denoted ‘U_6_’), in a 1:1 ratio at 310 K for 1 h prior to setting up the crystallization drop.

#### 2.5.2. Diffraction data collection and processing   

The crystallization conditions described above adequately protected *Aae* Hfq crystals against ice formation upon flash-cooling (presumably because of the MPD), making it unnecessary to transfer crystals to an artificial mother liquor/cryoprotectant. Crystals were harvested using nylon loops and flash-cooled with liquid nitrogen. Diffraction data were collected on beamlines 24-ID-E and 24-ID-C at the Advanced Photon Source (APS) for the apo and U_6_-bound crystal forms, respectively. Initial data-processing steps (indexing/integrating, scaling and merging reflections) were performed in *XDS* (Kabsch, 2010 ▸). Space-group assignments and unit-cell determinations utilized *POINTLESS* from the *CCP*4 suite (Winn *et al.*, 2011 ▸). The unit-cell parameters for the apo form (*P*1) were *a* = 63.46, *b* = 66.06, *c* = 66.10 Å, α = 60.05, β = 83.94, γ = 77.17° and those for the U_6_ co-crystals (*P*6) were *a* = *b* = 66.19, *c* = 34.21 Å.

#### 2.5.3. Structure solution, refinement and validation   

Initial phases for the diffraction data sets for both crystal forms were obtained *via* molecular replacement (MR). Specifically, the *Phaser* (McCoy *et al.*, 2007 ▸) software was used, with the *P. aeruginosa* (*Pae*) hexamer structure (PDB entry 1u1s; Nikulin *et al.*, 2005 ▸) as a search model for the phasing of both crystal forms (*Aae* and *Pae* Hfq share high sequence similarity; see Fig. 1 ▸). Note that initial phases for the *P*1 and *P*6 *Aae* crystal forms were obtained independently of one another, *i.e. via* parallel MR efforts. For the *P*1 (apo) form, with 12 monomers per unit cell (indicative of two hexamers), the calculated Matthews coefficient (*V*_M_) is 2.06 Å^3^ Da^−1^, corresponding to a solvent content of 40.21% by volume. For the *P*6 (U_6_-bound) form, only one monomer per asymmetric unit is feasible, with a *V*_M_ of 2.28 Å^3^ Da^−1^ and a solvent content of 46.08%. These and related characteristics of the diffraction data are summarized in Table 1 ▸.

After obtaining initial MR solutions in *Phaser*, the correct *Aae* Hfq amino-acid sequence was built and side chains were completed in a largely automated manner using the *AutoBuild* functionality in the *PHENIX* suite (Adams *et al.*, 2010 ▸). Individual solvent molecules, including H_2_O, MPD and Gnd, were added in a semi-automated manner (*i.e.* with visual inspection and manual adjustment) after the initial stages of refinement. Refinement of atomic positions, occupancies and atomic displacement parameters (ADPs), either as isotropic *B* factors or as full anisotropic ADPs, proceeded over several rounds in *PHENIX*. Some early refinement steps included simulated-annealing optimization of coordinates *via* molecular dynamics in torsion-angle space, as well as refinement of translation–libration–screw (TLS) parameters to account for anisotropic disorder of each subunit chain (one TLS group was defined per monomeric Hfq subunit). These steps yielded *R*_work_ and *R*_free_ values of 0.194 and 0.212 for the *P*1 data set and 0.212 and 0.223 for the *P*6 data set, respectively. The diffraction limits of the *P*1 and *P*6 forms, 1.49 and 1.50 Å, respectively, occupy an intermediate zone between the atomic resolution (*d* 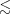 1.4 Å) and medium-resolution (*d* 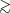 1.7 Å) limits whereupon clearer decisions can be made as to the treatment of *B* factors (Merritt, 2012 ▸). For instance, a relatively simple model (fewer parameters/atom), featuring individual isotropic *B* factors and one TLS group per chain, might be most justifiable at ∼1.6 Å, depending on the quality of the diffraction data, whereas a more complex *B*-factor model with a greater number of parameters, *e.g.* full anisotropic ADP tensors, *U_ij_*, one per atom, is likely to be statistically valid (and indeed advised) at resolutions better than ∼1.3 Å.

For both the *P*1 and *P*6 forms of *Aae* Hfq, a final *B*-factor model was chosen based on analyses of the data-to-parameter ratio (*i.e.* the number of reflections per atom), Hamilton’s generalized residual (Hamilton, 1965 ▸) and related criteria, as implemented in the *bselect* routine of the *PDB_REDO* code (Joosten *et al.*, 2012 ▸). The *P*1 and *P*6 data sets contained 16.5 and 17.5 reflections per atom, respectively, making the anisotropic refinement problem nearly twofold overdetermined; the unsupervised decision algorithm in *PDB_REDO* identified a fully anisotropic, individual *B*-factor model as being optimal. The structural models resulting from various ADP refinement strategies were assessed using the protein anisotropic refinement validation and analysis tool *PARVATI* (Zucker *et al.*, 2010 ▸). In the final refinement stages for both *Aae* Hfq crystal forms, *P*1 (*Z* = 12) and *P*6 (*Z* = 6), full anisotropic *B*-factor tensors were refined individually for virtually every atom. [A small fraction of atoms in both the *P*1 and *P*6 models were treated isotropically, *i.e.* by refining individual *B*_iso_ values; most of these atoms, selected based on per-atom statistical tests in *PDB_REDO*, were either water or heteroatoms (*e.g.* Gnd in *P*1, PEG in *P*6).] At no point in the refinement were NCS restraints or constraints imposed for the 12 subunits in the *P*1 cell. All refinement steps involving visual inspection and manual adjustment of the model were performed in *Coot* (Emsley *et al.*, 2010 ▸).

After the correct protein sequence had been built and refined against the *P*6 data set, at least two complete nucleotides of U_6_ RNA, including three phosphate groups, were clearly visible in α_A_-weighted difference electron-density maps (*mF*_o_ − *DF*_c_). Ribonucleotides were built into electron density using the *RCrane* utility (Keating & Pyle, 2010 ▸), after an initial round of refinement of coordinates, occupancies and individual *B* factors in *PHENIX*. Validation of the final structural models included (i) inspection of the Ramachandran plot *via* *PROCHECK* (Laskowski *et al.*, 1993 ▸), (ii) assessment of nonbonded interactions and geometric packing quality *via* *ERRAT* (Colovos & Yeates, 1993 ▸), (iii) analysis of sequence/structure compatibility *via* the profile-based method *Verify*3*D* (Eisenberg *et al.*, 1997 ▸) and, finally, (iv) detailed stereochemical/quality checks with the *MolProbity* software (Chen *et al.*, 2010 ▸). Final structure-determination and model-refinement statistics are provided in Table 2 ▸.

### 2.6. Sequence and structure analyses   

Sequences of verified Hfq homologs, drawn from diverse bacterial phyla, were selected for alignment and analysis against *Aae* Hfq. Here, we take ‘verified’ to mean that the putative Hfq homolog from the published literature has been identified *via* functional analysis or structural similarity (*e.g.* shown to adopt the Sm fold). Multiple sequence alignments were computed *via* two progressive-alignment codes: (i) the multiple alignment using fast Fourier transform method (*MAFFT*; Katoh & Standley, 2013 ▸) and (ii) a sequence-comparison approach using log-expectation scores for the profile function (*MUSCLE*; Edgar, 2004 ▸). The *Geneious* bioinformatics platform (Kearse *et al.*, 2012 ▸) was used for some data/project-management steps and tree-visualization purposes. Multiple sequence alignments (Fig. 1 ▸) were processed using *ESPript* (Gouet *et al.*, 1999 ▸) run as a command-line tool; the resulting PostScript source was then modified to obtain the final figures. Iterative *PSI-BLAST* (Camacho *et al.*, 2009 ▸) searches against sequences in the PDB were used to identify homologous proteins as trial MR search models. *Pae* Hfq, with 46% pairwise identity to *Aae* Hfq (across 97% query coverage), exhibited the greatest sequence similarity (∼63%, at the level of BLOSUM62) and was therefore chosen as the initial MR search model.

Structural alignments were performed using a least-squares fitting algorithm (McLachlan, 1982 ▸) implemented in *ProFit* (Martin & Porter, 2009 ▸). Multiple structural alignment of the 12 monomeric subunits in the apo form of *Aae* Hfq was used to create a mean reference structure, and each monomer was then aligned with this averaged reference. To assess three-dimensional structural similarity between each of the *n*(*n* −1)/2 distinct pairs of monomers, a pairwise distance matrix was constructed by computing main-chain r.m.s.d.s between subunits *i* and *j*, giving matrix element (*i*, *j*). Agglomerative hierarchical clustering was performed on this distance matrix using either the complete-linkage criterion or Ward’s variance-minimization algorithm with a Euclidean distance metric (Jain *et al.*, 1999 ▸); in-house code was written for these steps in both the R (within *RStudio*) and Python languages.

Residues were assigned to secondary-structural elements by a consensus approach *via* visual inspection in *PyMOL* as well as the automated assignment tools *DSSP* and *Stride*; the precise borders can differ between these codes by a residue or two. Normal-mode analyses of the *P*1 and *P*6 structures, taken as coarse-grained (C^α^-only) representations and treated as anisotropic network models (ANM), were performed with the *ProDy*/*NMWiz* (Bakan *et al.*, 2011 ▸) plugin to *VMD* (Humphrey *et al.*, 1996 ▸). The Hessian matrix of the ANM was built using default parameters for the force constant (γ = 1) and pairwise interaction cutoff distance (15 Å). Of the 3*N* − 6 nontrivial modes, displacements along the softest ∼20 vibrational modes, which correspond to low-frequency/high-amplitude collective motions, were visually inspected in *VMD*. Other structural analyses (*e.g.* Fig. 6*a*) entailed computing the principal axes of the moment of inertia tensor and the best-fit plane to three-dimensional structures (in the sense of linear least squares); the latter task utilized a previously described singular value decomposition code (Mura *et al.*, 2010 ▸), and all other structural analysis tasks employed in-house code written in Python or as Unix shell scripts. Nucleic acid stereochemical parameters and conformational properties, *e.g.* the values of glycosidic torsion angles and sugar pucker phase angles of the U_6_ RNA, were analysed and calculated with *DSSR* (Lu *et al.*, 2015 ▸). Surface-area properties, such as solvent-accessible surface area (SASA) and buried surface area (BSA or ΔSASA), were calculated as averages from five approaches: (i) Shrake and Rupley’s ‘surface-dot’ counting method (Shrake & Rupley, 1973 ▸), as implemented in *AREAIMOL*, (ii) the classic Lee and Richards ‘rolling-ball’ method (Lee & Richards, 1971 ▸), available in *NACCESS*, (iii) the ‘reduced surface’ analytical approach of *MSMS* (Sanner *et al.*, 1996 ▸), and the more approximate (point-counting) methods from the structural analysis routines available in (iv) *PyMOL* and (v) *PyCogent* (Cieślik *et al.*, 2011 ▸).

All molecular-graphics illustrations in Figs. 5–8 and Supplementary Figs. S3–S6 were created in *PyMOL*, with the exception of Supplementary Figs. S4(*e*) and S4(*f*) (created in *VMD* and rendered with *Tachyon*). *LigPlot*+ (Laskowski & Swindells, 2011 ▸) was used in creating schematic diagrams of interatomic contacts, as in Fig. 8. Many of the scientific software tools were used as SBGrid-supported applications (Morin *et al.*, 2013 ▸).

## 3. Results   

The organism *A. aeolicus* belongs to the taxonomic order Aquificales, in the phylum Aquificae, within what may be the most phylogenetically ancient and deeply branching lineage of the Bacteria. Thus, this species offers a potentially informative context in which to examine the evolution of sRNA-based regulatory systems, such as those built upon Hfq. The *Aae* genome contains an open reading frame with detectable sequence similarity to characterized Hfq homologs (*e.g.* from *E. coli* and other proteobateria), and an RNomics/deep-sequencing study has shown that, upon heterologous expression in the γ-proteobacterium *Salmonella enterica*, this putative Hfq homolog can immunoprecipitate host sRNAs (Sittka *et al.*, 2009 ▸). Sequence analysis confirms that this putative Hfq can be identified *via* database searches (Fig. 1 ▸), and that this homolog exhibits enhanced residue conservation at sequence positions that correspond to the three RNA-binding sites on the surface of Hfq, proximal, distal and lateral rim, denoted in the consensus line in Fig. 1 ▸. As the first step in our crystallo­graphic studies, we cloned, expressed and purified recombinant *Aae* Hfq: in these initial experiments, *Aae* Hfq generally resembled hitherto characterized Hfq homologs in terms of biochemical properties (*e.g.* resistance to chemical and thermal denaturation, and hexamer formation).

### 3.1. Cloning, expression, purification and initial biochemical examination of *Aae* Hfq   

Recombinant, wild-type *Aae* Hfq was successfully cloned, overexpressed and purified from *E. coli*, as confirmed by various biochemical and biophysical data, including SDS–PAGE gels (Supplementary Fig. S1) and MALDI-TOF mass spectra of the native protein (Fig. 2 ▸ *a*). The 6×His-tagged *Aae* Hfq is 100 amino acids in length, with a molecular weight of 11 365.0 Da and a predicted isoelectric point of 9.69; the working *Aae* Hfq construct, obtained *via* proteolytic removal of the tag (Supplementary Fig. S1*a*), is 83 amino acids in length (9482.9 Da, pI = 9.45). The expected mass computed from the amino-acid sequence is in close agreement with that experimentally characterized by MALDI–TOF, indicating successful (complete) removal of the affinity tag (Fig. 2 ▸ *a*) at position G^−2^ (residue numbering is such that the wild-type methionine is M^1^, as indicated in Supplementary Fig. S1*a*).

Initial *Aae* Hfq purification efforts were hindered by nucleic acid contaminants. Specifically, purified protein samples exhibited *A*_260_/*A*_280_ absorbance ratios of ∼1.65, indicative of co-purifying nucleic acids (De Mey *et al.*, 2006 ▸; Patterson & Mura, 2013 ▸); this problem is perhaps unsurprising given the known affinity of Hfq for nucleic acids, combined with the particularly high pI of *Aae* Hfq. By applying systematic colorimetric assays (Patterson & Mura, 2013 ▸) to *Aae* Hfq samples with high *A*_260_/*A*_280_ ratios (Supplementary Fig. S2*a*), we found that the co-purifying nucleic acids are likely to comprise a heterogeneous pool of RNAs with lengths between ∼100 and ∼200 nucleotides (Supplementary Fig. S2*b*). Early experiments using anion-exchange chromatography revealed that nucleic acid-bound Hfq would elute at three distinct ionic strengths (in a linear salt gradient), and each peak appeared to contain a population of nucleic acids that varied in length, both within one peak and between the three peaks (data not shown). To obtain well defined, well behaved apo *Aae* Hfq samples for downstream RNA-binding assays, crystallization trials *etc.*, relatively high concentrations (∼6 *M*) of guanidinium were added to the cell lysates, the aim being to dissociate spurious Hfq-associated nucleic acids. Inclusion of Gnd in the purification workflow (see §2.1) yielded samples with improved *A*_260_/*A*_280_ ratios (∼0.8), suggesting that nucleic acid contamination had been at least partly alleviated (pure protein samples generally have an *A*_260_/*A*_280_ of ∼0.7, and *E. coli* Hfq samples with an *A*_250_/*A*_274_ of ∼0.8 have been reported to have trace nucleic acid contamination; Updegrove *et al.*, 2010 ▸). Notably, the Gnd denaturant did not appear to unfold or disrupt the oligomerization properties of *Aae* Hfq based on various observations; for instance, a discrete band corresponding to the hexameric assembly persisted in SDS–PAGE gels of Gnd-treated samples (Supplementary Fig. S1*b*).

**Figure 3:**
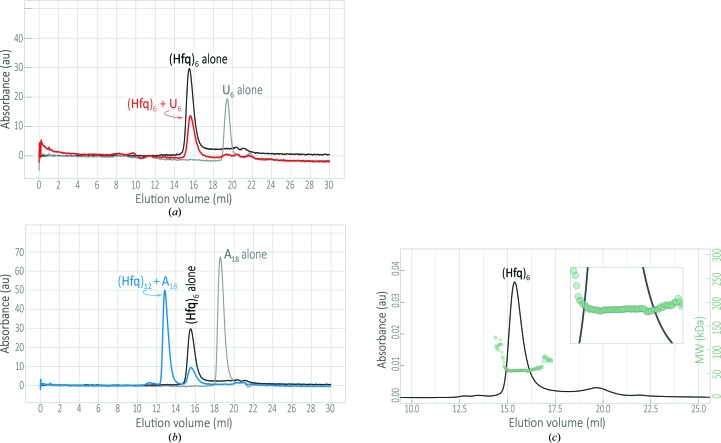
The solution-state distribution of *Aae* Hfq oligomers shifts in the presence of short RNAs. Elution profiles are shown for analytical size-exclusion chromatography of *Aae* Hfq samples incubated with either (*a*) U_6_ or (*b*) A_18_ RNAs [specifically, 250 µ*M* *Aae* Hfq was incubated in a 1:1(*v*:*v*) ratio with 50 µ*M* of either U_6_ or A_18_ RNA]. The elution of *Aae* Hfq was detected *via* the absorbance at 280 nm (*A*_280_), and RNA and Hfq·RNA complexes were monitored at *A*_260_. While putative Hfq⋯U_6_ interactions do not appear to shift the oligomeric state, as indicated by the close alignment of the black (Hfq alone) and red (Hfq·U_6_) peaks in (*a*), Hfq interactions with A_18_ do shift the oligomeric species towards a higher-order state [blue arrow in (*b*), denoting apparent dodecamers]. This shift could correspond to the simultaneous binding of A_18_ to two Hfq hexamers, potentially *via* two modes: (i) as an (Hfq)_6_·A_18_·(Hfq)_6_ ‘bridged’ complex or (ii) as A_18_ bound to one of the two distal faces that would be exposed on an independently stable (Hfq_6_)_2_ double-ring dodecamer. These two models cannot be distinguished *via* AnSEC. (*c*) To verify the molecular weight of the *Aae* Hfq elution peak, the protein was analysed *via* SEC fractionation followed by multi-angle static light-scattering and refractive-index measurements. The SEC elution profile (black trace) is taken as the absorbance at 280 nm. Light-scattering and refractive-index data can be used to compute molar masses, and the open circles shown here (semi-transparent green) are the molar-mass distribution data [*i.e.* masses (in kDa) as a function of elution volume]. The weight-averaged molecular weight, *M*_w_, of the Hfq sample is computed for the entire peak from this distribution, and the scale is given by the vertical axis on the right-hand side (green numbers; note that this scale applies to the main plot, not the inset). The apparent *M*_w_ that was computed, 58.75 kDa, corresponds to a hexameric assembly of *Aae* Hfq.

As an initial assessment of its self-assembly properties and oligomeric states in solution, purified *Aae* Hfq was examined by analytical size-exclusion chromatography (Figs. 3 ▸ *a* and 3 ▸ *b*, black traces). The protein elutes as a single, well shaped peak, with no apparent splitting, broadening, shouldering, tailing *etc*. However, the location of this peak is unexpected: the elution volume of the peak gives a molecular weight (MW) of ∼37 kDa, rather than the ∼57 kDa expected for an *Aae* Hfq hexamer. This apparent MW, obtained using a standard curve as described in §2.3, could indicate a tetrameric assembly, for which the MW is calculated to be 37.9 kDa. Shape-dependent deviations from ideal migration properties would be expected to give an (Hfq)_6_ species that migrates faster, not slower, than anticipated based purely on MW, given the larger effective hydrodynamic radius of a toroidal hexamer (*versus* the roughly globular standards used to calibrate our column elution volumes). However, favorable protein–resin inter­actions would tend to retard the migration of an *Aae* Hfq oligomer, leading to a smaller apparent MW species. Given the highly basic pI, and the resultant charge on *Aae* Hfq at near-neutral pHs, we suspect that the low MW estimate from AnSEC stems from protein–resin interactions, electrostatic or otherwise; spurious *Aae* Hfq retention was also observed in experiments with other, unrelated chromatographic resins. Note that nonspecific protein adsorption to SEC resins was first documented long ago (Belew *et al.*, 1978 ▸) and has been reviewed by Arakawa *et al.* (2010 ▸).

The aberrant AnSEC elution behavior prompted us to assay the *Aae* oligomeric state by alternative means. SEC coupled with multi-angle light scattering (MALS) showed that the *Aae* Hfq eluting at this peak position corresponds to a hexamer, with a weight-averaged molecular weight, *M*_w_, of 58.75 kDa (Fig. 3 ▸ *c*). A plot of the molar-mass distribution (Fig. 3 ▸ *c*, green circles) exhibits uniform values across this *Aae* Hfq peak (Fig. 3 ▸ *c*, inset), indicating that this region of the eluted sample is monodisperse. *Aae* Hfq monomers were found to be susceptible to chemical cross-linking with formaldehyde, as analysed by MALDI-TOF MS (Fig. 2 ▸). The main peak in the mass spectrum of this sample (Fig. 2 ▸ *b*) corresponds to a hexamer (57 498.0 Da from MS *versus* 56 897.4 Da from the sequence); a second peak, near 115 kDa, corresponds to within 1.5% of the MW of a dodecameric assembly. Some Sm and Hfq orthologs have been found to assemble into stacked double rings and other higher-order species, based on analytical ultracentrifugation and light-scattering data (Mura, Kozhukhovsky *et al.*, 2003 ▸; Mura, Phillips *et al.*, 2003 ▸; Dimastrogiovanni *et al.*, 2014 ▸), electron microscopy (Arluison *et al.*, 2006 ▸; Mura, Kozhukhovsky *et al.*, 2003 ▸), gel-shift assays and other approaches; however, an integrated experimental analysis, using multiple independent methodologies on the same Hfq system, strongly suggests that the *E. coli* (Hfq)_6_·RNA binding stoichiometry is predominantly 1:1 (Updegrove *et al.*, 2011 ▸).

### 3.2. Characterization of RNA binding by *Aae* Hfq in solution   

To evaluate putative RNA interactions with *Aae* Hfq, solution-state binding interactions between *Aae* Hfq and either U_6_ or A_18_ (unlabeled) RNAs were examined *via* analytical size-exclusion chromatography. RNAs that are U-rich (*e.g.* U_6_) or A-rich [*e.g.* harboring an (A–A–N)*_n_* motif] are known to bind at the proximal and distal faces, respectively, of Hfq homologs from Gram-negative species. We found that U_6_ RNA binds *Aae* Hfq in solution, based on comparisons of the following elution profiles (Fig. 3 ▸ *a*): (i) Hfq only (black trace, detected *via* absorbance at 280 nm), (ii) U_6_ only (gray, monitored at 260 nm) and (iii) an Hfq and U_6_ mixture (red, 260 nm). In sample (iii), the Hfq + U_6_ mixture, note the absence of a U_6_ RNA peak near 19.5 ml (Fig. 3 ▸ *a*, gray) and a concomitant peak shift to a position centered at the Hfq-only trace, indicating saturated binding of the RNA. Properties of the elution profiles for samples (i) and (iii), specifically, no shift in the peak position and no alteration of the bilateral symmetry of the peak (no tailing, shouldering *etc.*), suggest that the addition of U_6_ does not alter the distribution of the apparent oligomeric states of *Aae* Hfq.

In contrast to the U_6_ behavior, adding A_18_ RNA to an *Aae* Hfq sample does appear to shift the Hfq oligomeric state to a higher-order species (Fig. 3 ▸ *b*, blue trace, major peak) that coexists with the usual hexamer (blue trace, minor peak). This newly appearing, A_18_-induced species is hydrodynamically larger than (Hfq)_6_, as it elutes far earlier than does Hfq in the Hfq-only sample (black trace); the higher-order entity appears to correspond to an *Aae* Hfq dodecamer. This was further verified based on the *M*_w_ determined *via* SEC-MALS experiments performed in parallel, which agrees to within 0.5% with the ideal *M*_w_ of an [(Hfq)_6_]_2_·A_18_ complex (Supplementary Fig. S3). Also, note that the Hfq+A_18_ trace is devoid of a peak at the A_18_-only position (*i.e.* no peak in the blue trace near the ∼18.5 ml peak location in the gray trace), indicating that binding has saturated with respect to A_18_.

**Figure 4:**
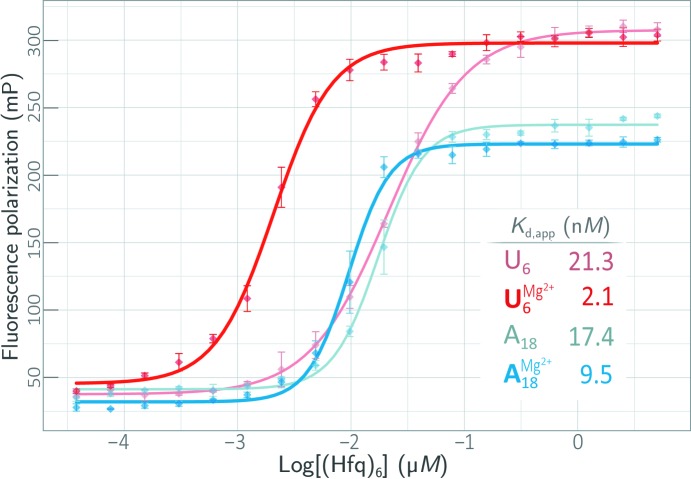
High-affinity binding of *Aae* Hfq to A-rich and U-rich RNAs, with variable Mg^2+^ dependencies. Binding was quantified *via* fluorescence polarization assays using 5 n*M* FAM-U_6_ (red) or FAM-A_18_ (blue) and varying concentrations of Hfq, either in the absence (thin lines) or presence (thick lines) of 10 m*M* MgCl_2_. For each binding reaction, data from three replicates (standard errors given by vertical bars) were fitted using a four-parameter logistic function to model the sigmoidal binding isotherm; nonlinear fits were also performed with an alternative model, accounting for receptor depletion but neglecting cooperativity (§[Sec sec2.4]2.4 and Supplementary Fig. S4). The computed binding constants are given (inset) in terms of the (Hfq)_6_ concentration, as the stoichiometry of all characterized Hfq·RNA complexes, as well as the structural results reported here, suggest that a hexamer is the active/functional unit. The addition of Mg^2+^ increases the binding affinity for both FAM-U_6_ and FAM-A_18_, albeit with a greater influence for the U-rich (proximal site-binding) RNA. Significant binding was not detected for a shorter A-rich (FAM-A_6_) or C-rich (FAM-C_6_) ssRNA.

To further quantify the interactions of Hfq with U-rich and A-rich RNAs, the binding affinities of *Aae* Hfq for 5′-FAM-labeled RNA oligoribonucleotides were determined *via* fluorescence polarization (FP) assays (Fig. 4 ▸, Supplementary Fig. S4). FAM-U_6_ and FAM-A_18_ probes were taken as proxies for U-rich and A-rich ssRNAs, enabling us to assay the strength of *Aae* Hfq⋯RNA interactions with these prototypical A/U-rich RNAs (for brevity, we refer to these RNAs as simply ‘U_6_’ and ‘A_18_’ if the FAM is obvious from the context). Both U_6_ and A_18_ were found to bind *Aae* Hfq with similarly high affinities: using a full nonlinear (logistic function) treatment of the sigmoidal binding isotherm given by equation (1), the nanomolar-scale apparent dissociation constants (*K*_d,app_) are 21.3 n*M* for U_6_ and 17.4 n*M* for A_18_ (Fig. 4 ▸, thin, lighter-color traces). The sigmoidal shape of these binding curves indicates positive cooperativity, and the Hill coefficients were calculated to be 1.3 and 2.2 for U_6_ and A_18_, respectively. The inclusion of 10 m*M* Mg^2+^ in the binding reaction enhanced the U_6_-binding affinity by an order of magnitude, yielding a *K*_d,app_ of 2.1 n*M* (Fig. 4 ▸; red, thicker trace) with a Hill coefficient of 1.7; the A_18_-binding affinity also increased in the presence of Mg^2+^, although by only twofold, to a *K*_d,app_ of 9.5 n*M* (blue, thicker trace) with a Hill coefficient of 2.4.

Because the apparent *K*_d_ values for U_6_ and A_18_ binding were found to be in the low nanomolar range, depletion of the Hfq receptor must be accounted for near the lower Hfq concentration range sampled in our binding assays (approximately, the nanomolar range; Fig. 4 ▸). Receptor-depletion phenomena can lead to spuriously high values of *K*_d,app_ as computed from nonlinear regression against FP data, as detailed in §[Sec sec2.4]2.4. Thus, to assess the impact of receptor depletion, we also performed a nonlinear least-squares fit of a three-parameter form of the classic binding isotherm (§2.4) against the FP binding data. This model [equation (2) in §2.4] yielded the results shown in Supplementary Fig. S4, with *K*_d_ values that were indeed ∼20–40% lower in magnitude than those calculated by fitting with the full sigmoidal/logistical model (*i.e.* using equation 1). Note, however, that this three-parameter model assumes a Hill coefficient fixed at unity and does not account for the aforementioned positive cooperativity that we detect in *Aae* Hfq⋯RNA binding (see the discussion of the d*x* parameter in §[Sec sec2.4]2.4). Also, note that the 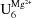 and 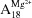 Hfq-binding reactions, which had the lowest *K*_d_ values (2.1 and 9.5 n*M*, respectively) of the four systems shown in Fig. 4 ▸ and Supplementary Fig. S4, were also the two systems that featured the greatest discrepancy in the *K*_d,app_ computed *via* equation (1) (includes cooperativity, neglects depletion) *versus* equation (2) (neglects cooperativity, accounts for depletion); this is a reassuring finding in terms of a depletion model for our *Aae* Hfq·RNA system, as the discrepancies that arise from receptor depletion become disproportionately greater at lower *K*_d_ values. Finally, we note that no significant binding was detected between *Aae* Hfq and either FAM-A_6_ or FAM-C_6_ (data not shown).

### 3.3. Crystal structures of *Aae* Hfq monomers and oligomers, and their lattice packing   

Crystals of *Aae* Hfq were readily obtained in multiple forms, including hexagonal plates and small, birefringent parallelepiped habits (Supplementary Fig. S1*c*). At least three distinct morphologies could be identified, which we denote (i) a ‘*P*1 form’ (apo Hfq, without RNA), (ii) a ‘*P*6 form’ (with RNA; see §[Sec sec3.5]3.5) and (iii) a third form that is likely to belong to space group *P*3_1_ or *P*6_2_. Forms (i) and (ii) were well diffracting (Supplementary Fig. S1*d*), leading to the *P*1 and *P*6 structures reported here; the third form yielded diffraction data with potential pathologies, including translational pseudosymmetry or tetartohedral twinning, and its structure will be the subject of future work (K. A. Stanek & C. Mura, unpublished work). Initial *Aae* Hfq crystals were obtained with a crystallization reagent comprised of 0.1 *M* sodium cacodylate, 5%(*w*/*v*) PEG 8000, 40%(*v*/*v*) MPD; inclusion of the additive [Co(NH_3_)_6_]Cl_3_ at ∼10 m*M* in the final crystallization drop improved the specimen size and quality. These apo *Aae* Hfq crystals formed in space group *P*1, with unit-cell parameters *a* = 63.46, *b* = 66.06, *c* = 66.10 Å, α = 60.05, β = 83.94, γ = 77.17°. These dimensions are most consistent with *Z* = 10–12 monomers per cell, and a resolution-dependent probabilistic estimator for the Matthews coefficient (Kantardjieff & Rupp, 2003 ▸) gives a 12-mer as the second-highest peak; also, the *a* ≃ *b* ≃ *c* geometry is consistent with a model in which two Hfq hexameric rings, which generally measure ∼65 Å in diameter, stack atop one another in the cell.

The *P*1 *Aae* Hfq structure was refined to 1.49 Å resolution, with initial phases obtained by molecular replacement with a *Pae* Hfq hexamer search model (PDB entry 1u1s; Nikulin *et al.*, 2005 ▸). The *Pae* homolog was used because sequence analysis (Fig. 1 ▸) showed it to have the greatest sequence identity (>40%) to *Aae* Hfq. A promising molecular-replacement solution was readily identified, and side chains for the *Aae* Hfq sequence were initially built in an automated manner using *PHENIX*. As detailed in §2.5.3, the number of reflections per atom, as well as other diffraction data-quality statistics, prompted us to refine the atomic displacement parameters (ADPs) *via* treatment of the full, anisotropic *B*-factor tensor for essentially all non-H atoms (most of the isotropically treated exceptions were atoms of solvent molecules or small-molecule components of the crystallization buffer). Anisotropic treatment of individual ADPs began at a relatively late stage in the overall refinement workflow, and doing so noticeably improved the *R*_work_ and *R*_free_ residuals from 13.6 and 17.2%, respectively, before anisotropic treatment to 13.2 and 16.9%, respectively, after anisotropic treatment (Table 2 ▸). The final, refined *P*1 model was subjected to extensive validation and quality assessment, in terms of both the three-dimensional structure itself (*i.e.* atomic coordinates) as well as the patterns of *B* factors (*i.e.* anisotropic ADPs), as described in §2.5.3.

**Figure 5:**
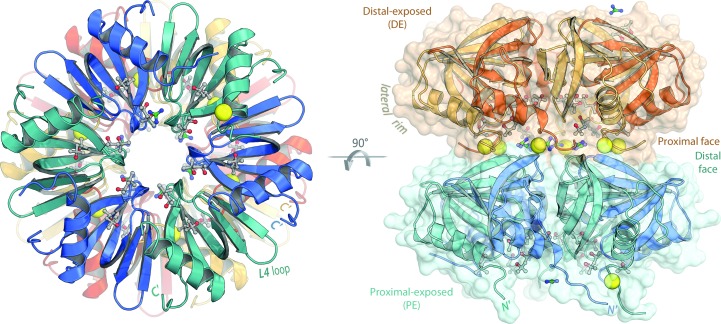
Crystal structure of *Aae* Hfq in the apo form, with head→tail stacking of hexameric rings. The apo form of *Aae* Hfq crystallized in space group *P*1 as a dodecameric assembly of hexamers stacked in a proximal→distal orientation in the lattice. Ribbon diagrams of the final, refined structure are shown here from perpendicular viewpoints. The proximal-exposed (PE) hexamer is colored blue and cyan, and subunits in the distal-exposed (DE) hexamer are colored alternatingly yellow and orange. Co-crystallizing molecules of MPD (gray C atoms) and Gnd (green C atoms) are shown in ball-and-stick representation, and Cl^−^ ions are rendered as yellow spheres scaled to the van der Waals radius. Note that many of the Gnd cations and Cl^−^ anions are coplanar, where they form a ‘salty’ layer at the ring interface (this is most clearly seen in the transverse view). Contacts between hexamers are mediated by the N-termini of the DE hexamer (top) and the loop L2/strand β2 regions of the PE hexamer (bottom); the approximate location of one of the lateral rim RNA-binding sites is labeled on the DE ring.

In addition to >400 solvent (H_2_O) molecules, the final *P*1 model also includes four PEG fragments, eight Gnd molecules, seven Cl^−^ ions and 25 MPD molecules (Table 2 ▸). Six each of the Gnd cations and chloride anions bind between the two Hfq rings, in identical positions with respect to the nearest protein subunit (*i.e.* in a sixfold-symmetric arrangement; Fig. 5 ▸); the other Gnd and Cl^−^ species occur at unremarkable locations. The PEG fragments bind in a concave region on the exposed face of the DE ring, *i.e.* on the distal surface of *Aae* Hfq (not shown in Fig. 5 ▸ for clarity). Notably, this moderately apolar pocket corresponds to the second A site in the (A–A–N)*_n_* recognition motif described above (§[Sec sec1]1). The cleft is formed between adjacent subunits (at the interfaces of chains *I*/*J*, *J*/*K*, *K*/*L* and *L*/*G*), and is well defined in *Aae* Hfq, with one of its walls formed by the phenolic ring of Tyr23 (homologous to *E. coli* Tyr25, which is crucial for A-rich RNA binding). The PEG fragments bind with similar poses in each of the four sites. Of the 25 MPD molecules, 24 occupy sixfold-symmetric positions near the proximal face of *Aae* Hfq (the remaining MPD is near the distal face of the DE ring). These 24 MPDs bind in a 2 × (6 + 6′) arrangement. Here, the ‘2’ denotes that a set of 12 MPDs binds identically to each of the two Hfq hexamers (*i.e.* the PE and DE rings in Fig. 5 ▸), and the prime in ‘6 + 6′’ indicates two distinct subsets of MPDs: one binds at the proximal RNA site of Hfq (below, and Fig. 7), while the other MPD is disposed near the α-helix on the proximal site, not far from the lateral rim.

**Figure 6:**
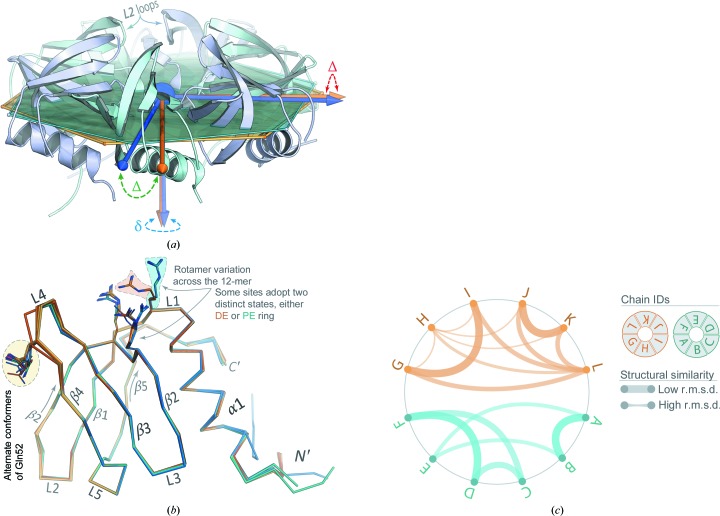
Structural variation across the *Aae* Hfq monomer (*P*6) and dodecamer (*P*1) crystal forms. At a gross structural level, the two Hfq rings in the head-to-tail dodecamer of the *P*1 crystal form (Fig. 5 ▸, axial view) appear to be related by a rigid-body rotation. The two rings, the proximal-exposed (PE) and distal-exposed (DE) hexamers, were brought, *via* pure rigid-body translation, to a common origin, indicated by the blue sphere in (*a*). Best-fit planes to each ring were then computed as described in §[Sec sec2.6]2.6 and shown here as semi-transparent hexagonal plates of either orange (DE ring) or cyan (PE ring) color. For clarity, the DE ring (orange/yellow in Fig. 5 ▸) is omitted in (*a*), and a couple of the L2 loops are labeled (in the PE ring) simply as a structural landmark. The three principal axes of the moment of inertia tensor are shown in either orange (DE ring) or blue (PE ring); large differences in the orientation of these principal axes are marked by green and red ‘Δ’ symbols, while a ‘δ’ symbol (blue) denotes smaller-scale differences. The rotation between the rings is clear from the relative disposition (Δ) of two of the principal axes. Furthermore, a small, but discernable, difference (δ) in the directions of the normal axes indicates a slight tilt between the rings; this direction would correspond to the sixfold axis in a perfectly symmetric double hexamer. A multiple structural alignment of the 12 subunits in the *P*1 cell (*b*) reveals little structural variation of the Sm core (shown as C^α^ backbone traces), while there are many examples of side-chain variability (as noted in the panel). The defining secondary-structural elements of the Sm fold (L1 loop, β1 strand *etc.*), as well as the termini, are labeled. The two regions of *Aae* Hfq that most extensively engage in interactions between rings (hexamer–hexamer contacts in Fig. 5 ▸), and in forming crystal contacts, are the L4 loops and the irregularly structured ∼5 residues at the N-terminus (preceding α1). These also are the two most variable regions in Hfq, both in terms of sequence length (and composition) as well as three-dimensional structure, as seen in (*b*). The side-chain variability shown in (*b*) takes two forms: (i) alternate conformers that could be built for a single residue, such as the Gln52 example highlighted to the left, and (ii) rotameric variation for a single residue across the 12 subunits, such as the groups of three residues shown as sticks near the top of (*b*). In many instances of the latter case, the 12 residue states clustered into two groups, corresponding to the DE or PE hexamer. In the diagram in (*c*), the Hfq subunits in *P*1, labeled by chain ID, are evenly spaced about a circle; arcs are drawn between the most structurally similar pairs of subunits, with the line thickness inversely scaled by the r.m.s.d. for the given pair. For clarity, not all ∼*n* ^2^ edges are shown here, but rather only at the levels of subunit pairs and triples (*i.e.* the deepest and second-deepest levels of leaf-nodes in the full dendrogram of Supplementary Fig. S5*c*). This result, from hierarchical clustering on backbone r.m.s.d.s, shows that pairs of monomers within a given hexamer are structurally more similar to each other than are pairs between hexamers (chains *A*→*F* comprise the PE ring and chains *G*→*L* comprise the DE ring).

The overall three-dimensional structure of the *Aae* Hfq monomer (Fig. 5 ▸) is that of the Sm fold, as anticipated based on sequence similarity and the efficacy of MR in phasing the diffraction data. In particular, the N-terminal α-helix is followed by five highly curved β-strands arranged as an antiparallel β-sheet. The secondary-structural elements (SSEs), shown schematically in Fig. 1 ▸, are labeled in the three-dimensional structure of Fig. 6 ▸(*b*). The precise SSE boundaries in *Aae* Hfq, computed with *Stride*, are residues 5–16 (α1), 19–24 (β1), 29–38 (β2), 41–46 (β3), 49–54 (β4) and 58–63 (β5); the same ranges are obtained with *DSSP*, save that the *DSSP* criteria make Phe37 (not Asp38) the end of the most curved strand (β2). Most of the β-strands in *Aae* Hfq are delimited by loops that adopt various β-turn geometries (including types I, II′, IV and VIII), with the exception of a short 3_10_-helix (residues 55–57) between β4 and β5. These loops contain many of the RNA-contacting residues of Hfq (see below) and, as labeled in Figs. 1, 5, 6 and 9, we denote these linker regions as L1→L5. Noncovalent interactions between Hfq monomers include van der Waals contacts and hydrogen bonds between the backbones of strand β4 of one subunit and β5* of the adjacent subunit, effectively extending the β-sheet across the entire toroid; these enthalpically favorable interatomic contacts are likely to facilitate self-assembly of the hexamer. (Unless otherwise stated, asterisks denote an adjacent Hfq subunit, be it related by crystallographic symmetry or otherwise.) Residues 1→68 of the native *Aae* Hfq sequence could be readily built into electron-density maps for each monomer in the asymmetric unit, thus providing a structure of the N-terminal region of Hfq as well as the entire Sm domain; note that the N-terminal tail, illustrated for the apo/*P*1 structure in Fig. 5 ▸ (bottom right) and Fig. 6 ▸(*b*), was unresolved in many previous Hfq structures. Most of the *Aae* Hfq C-terminal residues 70→80 were not discernible in electron density and are presumably disordered.

### 3.4. The apo form of *Aae* Hfq   

While neither NCS averaging, nor any NCS constraints or restraints, were applied at any point during the phasing and refinement of *Aae* Hfq in the apo form, the 12 monomers in the *P*1 cell are virtually indistinguishable from one another (Figs. 6 ▸ *a* and 6 ▸ *b*, Supplementary Fig. S5), at least at the level of protein backbone structure (there are side-chain variations). The mean pairwise main-chain r.m.s.d. averaged over all monomer pairs in the *P*1 cell lies below 0.3 Å; this low value is also evident in the magnitude of the ordinate scale of the structural clustering dendrogram in Supplementary Fig. S5(*c*). To systematically compare structures, a matrix of r.m.s.d.s was constructed from all pairwise subunit alignments. Agglomerative hierarchical clustering on this distance matrix (Supplementary Fig. S5*c*) reveals that the subunits partition into two low-level (root-level) clusters so as to recapitulate the natural (structural) ordering found in the crystal: that is, chains *A*→*F* cluster together (as the proximal-exposed, or PE, ring in Fig. 5 ▸), and likewise chains *G*→*L* form a second group (the distal-exposed, or DE, ring). This finding is illustrated in Fig. 6 ▸(*c*), which conveys the degree of three-dimensional structural similarity as a circular graph wherein the width of an edge between two chains is inversely scaled by their pairwise r.m.s.d.

At the *Aae* Hfq monomer level, the greatest structural variation occurs among the N-termini and the L4 loop region between β3→β4; apart from the termini, loop L4 (Fig. 6 ▸ *b*) is the most variable region in most known protein structures from the Sm superfamily. The conformational heterogeneity in the termini and loops of *Aae* Hfq stems, at least partly, from differing patterns of interatomic contacts for different sub­units at the levels of monomers, hexamers and dodecamers in the overall *P*1 lattice. The patterns of conformational heterogeneity are clear when the dodecameric structure is visualized as a cartoon, with the diameter of the backbone tube scaled by the magnitude of per-atom *B*_eq_ values (this derived quantity, computed from the trace of the full anisotropic ADP tensor, is taken as an estimate of the true *B*_iso_ values that would result from refinement of an isotropic model); such renditions are shown in Supplementary Figs. S6(*a*) and S6(*b*) for the *P*1 and *P*6 structures, respectively. Analogously, Supplementary Figs. S6(*c*) and S6(*d*) provide thermal ellipsoid representations of the patterns of variation in anisotropic ADPs across the *P*1 dodecamer and the *P*6 monomer. In both sets of depictions, Supplementary Figs. S6(*a*) and S6(*b*), and Figs. S6(*c*) and S6(*d*), colors are graded by the magnitude of per-atom *B*_eq_ values from low (blue) to medium (white) to high (red). To initially assess the relative contributions of static disorder (*e.g.* variation in rotameric states across subunits) and dynamic disorder (*e.g.* harmonic breathing modes and other collective/global motions) in variable regions such as loop L4 and the termini, a normal-mode analysis was performed on a coarse-grained representation of the *Aae* Hfq structures, using an anisotropic network model of residue interactions (see §2.6). Illustrative results for the dodecamer and monomer are shown in Supplementary Figs. S6(*e*) and S6(*f*), respectively. The pattern of normal-mode displacements for both the dodecamer and monomer do not implicate loop L4 in any especially high-amplitude, low-frequency modes (Supplementary Fig. S6*f*), suggesting that the increased ADPs (elevated *B*_eq_ values) of L4 stem more from static disorder rather than any particular dynamical process involving this loop region (although anharmonic dynamics remain possible). The dodecamer calculation does reveal a significant harmonic mode corresponding to antisymmetric rotation of the two Hfq rings with respect to one another (PE ↺, DE ↻; Supplementary Fig. S6*e*). This result is consistent with our observation that the only large-scale (dodecamer-scale) structural difference between the two rings is a slight rotation of one relative to the other (Fig. 5 ▸, left) *versus*, for instance, a rigid-body tilt (Fig. 6 ▸ *a*, Supplementary Figs. S5*a* and S5*b*).

At the Hfq ring and supra-ring levels, the refined *P*1 structure reveals an *Aae* Hfq dodecamer consisting of two hexameric rings stacked in a head→tail orientation (Fig. 5 ▸). Propagated across the lattice, this arrangement gives cylindrical tubes with a defined polarity. The tubes run along the crystallographic **a** axis, and their lateral packing yields near-sixfold symmetry along this direction; a slight translational shift of the dodecamers in adjacent unit cells, in the plane perpendicular to **a**, causes the rings to be slightly offset with respect to the lattice tubes (the tubes are not perfectly cylindrical, insofar as the sixfold axis of an individual Hfq ring is not coaxial with the principal axis of its parent tube). In the dodecamer, the distal face of one Hfq ring is exposed (termed the DE ring), while the other ring features a proximal-exposed face (the PE ring; Fig. 5 ▸, right). The N-termini of the DE hexamer contact the L2-loop/β2-strand region of the PE ring, as illustrated in Fig. 5 ▸ (the L2 loops mark the beginning of strand β2; see the label in Fig. 6 ▸ *a*). As is apparent in the axial view of Fig. 5 ▸ (left), one ring is slightly rotated relative to the other. Geometric analysis of this rotation (denoted ‘Δ’ in Fig. 6 ▸ *a*), as well as other rigid-body transformations relating the two rings (Supplementary Figs. S5*a* and S5*b*), shows that the sixfold symmetry axes of the rings in the dodecamer are not perfectly parallel: a slight tilt occurs between the rings (‘δ’ in Fig. 6 ▸ *a*). This tilt appears to stem largely from structural differences in the N-terminal regions (Supplementary Fig. S5). Consistent with these observations, the set of six N-terminal regions of the DE ring (which mediate ring–ring interactions within a dodecamer) exhibit slightly higher *B*_eq_ values and greater conformational variability than do the six N-termini of the PE ring (which mediate dodecamer⋯dodecamer contacts between unit cells), as can be seen in Supplementary Fig. S6(*a*).

Noncovalent molecular interactions between the proximal⋯distal faces mediate the association of Hfq rings into a dodecamer, and a slightly altered (translationally shifted) version of these same energetically favorable interactions stitches together the dodecamers into a set of crystal lattice contacts in the *P*1 form of *Aae* Hfq. Notably, a proximal→distal stacking geometry is also the chief mode of ring association in the *Aae* Hfq *P*6 lattice. *Aae* Hfq dodecamers clearly occur in the *P*1 lattice, with a substantial amount of buried surface area (BSA) defining the ring–ring interface (Fig. 5 ▸). Specifically, 3663 ± 244 Å^2^ of SASA is occluded between the PE and DE hexamers in the PE–DE complex. Note that this quantity is reported as a total BSA = ASA_PE_ + ASA_DE_ − ASA_PE–DE_, where ASA_*i*_ is the ASA of species *i*, rather than as the per-subunit value (which would be given by half of the above expression, were we to assume a perfectly twofold symmetric interface); also, note that this mean ± standard deviation is reported from the results of five different surface-area calculation approaches, as described in §2.6.

### 3.5. Crystal structure of *Aae* Hfq bound to U_6_ RNA   

**Figure 7:**
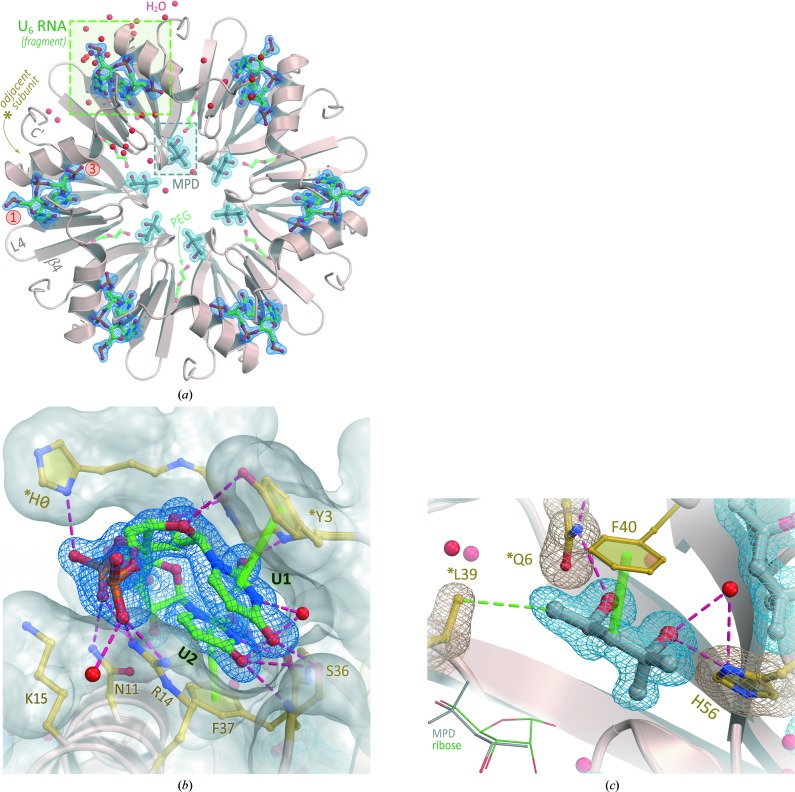
Crystal structure of *Aae* Hfq with U-rich RNA bound at the lateral rim. The asymmetric unit of the *P*6 form contains a single Hfq subunit, shown as a tan-colored ribbon diagram (*a*), in addition to 36 H_2_O molecules (red spheres), a molecule of PEG (lime-colored C atoms), a molecule of MPD (gray C atoms) and one molecule of U_6_ RNA (green C atoms). Nonprotein atoms are shown in ball-and-stick representation using CPK colors (except as noted above for C atoms). Expansion of the asymmetric unit to the full *P*6 cell gives an intact Hfq hexamer, shown on the proximal face in (*a*). The meshes delimit the 2*mF*_o_ − *DF*_c_ electron-density map, contoured at 1.5σ and shown only in the regions of RNA (dark blue) or MPD (light blue). The fragment of U_6_ that could be unambiguously built into electron density contained two complete uridines and the 5′ phosphate moiety of the next residue; the path of this RNA strand is denoted by a red-circled 1 and 3 for the ribonucleotides, from 5′ to 3′. Unexpectedly, U_6_ nucleotides were found on the outer rim of *Aae* Hfq, in a position analogous to the lateral site of other Hfqs (*b*), while a molecule of MPD occupied the U-rich binding pore as shown in (*c*). This magnified view (*b*) of the lateral site [same color scheme as (*a*)] shows the RNA-contacting residues (labeled) in greater detail; asterisks distinguish residues from the N-termini of a neighboring subunit, as also indicated in (*a*). Electron-density maps such as this one were readily interpretable as RNA (see also Supplementary Fig. S7). The magenta dashed lines (hydrogen bonds) and semi-transparent green cylinders (π-stacking interactions) indicate enthalpically favorable Hfq⋯RNA contacts. Most such contacts are mediated by both backbone and side-chain atoms of *Aae* Hfq, as well as the nucleobase and phosphodiester groups of the RNA; the ribose rings project outward from the cleft and interact with Hfq more sparsely. (*c*) MPD binds at the pore and mimics the Hfq⋯uridine contacts found at the proximal RNA-binding site in some Hfq homologs. Contacts denoted by magenta dashed lines identically match the contacts to a uridine nucleotide in other Hfq structures containing U-rich RNA (see also Supplementary Fig. S8). The green line indicates a van der Waals contact between Leu39 and MPD, and the green cylinder denotes another apolar interaction between *Aae* Hfq and MPD; this latter contact would presumably be replaced by a π-stacking interaction between Phe40 and a U base, were a U-rich RNA (rather than MPD) bound at the proximal site.

Upon co-crystallization with U_6_ RNA, a second, distinct *Aae* Hfq crystal form was discovered. These crystals could be indexed in space group *P*6, with unit-cell parameters *a* = *b* = 66.19, *c* = 34.21 Å. In this form, the cell geometry, solvent content and molecular mass of *Aae* Hfq are only compatible with a single Hfq monomer per asymmetric unit; based on known Hfq structures, the crystallographic sixfold axis was presumed to generate intact hexamers, such as that shown in Fig. 7 ▸(*a*). Specifically, co-crystallization of *Aae* Hfq with this model uridine-rich RNA was achieved by incubating purified Hfq samples with 500 µ*M* U_6_ RNA prior to crystallization trials. The complex crystallized in 0.1 *M* sodium cacodylate, 5%(*w*/*v*) PEG 8000, 40%(*v*/*v*) MPD, and the denaturant compound Gnd was found to be an effective additive (Supplementary Table S2). The crystal structure of the *Aae* Hfq·U_6_ RNA complex was refined to 1.50 Å resolution (Fig. 7 ▸); we emphasize that the initial solution of this structure was achieved independently of the apo *P*1 form, *via* molecular replacement, using *P. aeruginosa* Hfq as a search model.

Those residues that are crucial in forming the proximal (U-rich) RNA-binding pocket in *E. coli* Hfq and other Hfq orthologs, *i.e.* *E. coli* Hfq residues Gln8, Phe42, Lys56 and His57, are conserved in the *Aae* Hfq sequence (Fig. 1 ▸). This observation led us to anticipate that any bound U_6_ would be localized to the proximal pore region. Instead, a molecule of MPD, which served as a precipitant and cryoprotectant in our crystallization experiments (Supplementary Table S2), was found to occupy the proximal site of the hexamer, with the MPD hydroxyl groups hydrogen-bonded to the side chains of the His56 and *Gln6 residues of *Aae* Hfq (Fig. 7 ▸ *c*). In addition, the bound MPD makes van der Waals contacts with other conserved residues that line the proximal site, specifically *Leu39 and Phe40. During refinement of this structure, two nucleotides of the U_6_ RNA molecule, including the flanking 5′ and 3′ phosphates (the latter coming from the third U), were readily discernible in *mF*_o_ − *DF*_c_ difference electron-density maps (Supplementary Fig. S7). Rather than being bound at the proximal site, the uridine residues of U_6_ occupied a cleft formed between the N-terminal α-helix and strand β2, in a position located roughly near the outer (‘lateral’) rim of the *Aae* Hfq toroid (Figs. 7 ▸ *a* and 7 ▸ *b*). Notably, processing and reduction of the diffraction data (collected from *P*6-form crystals) in *P*1 yielded similar electron density for the RNA at each lateral binding pocket in the hexamer (Supplementary Fig. S7).

### 3.6. RNA binding at the outer rim of the *Aae* Hfq hexamer: structural details   

The *Aae* Hfq·U_6_ structure reveals a lateral RNA-binding pocket that accommodates two uridine nucleotides. The N-terminal α-helix primarily contacts the phosphodiester and ribose groups, and the β2 strand interacts mostly with the uracil bases (Figs. 7 ▸ *a*, 7 ▸ *b* and 8 ▸ *a*). As a consequence of this RNA-binding geometry, both nucleotides that were fully built into electron density (U1 and U2) are held in a bridging, *anti* conformation (χ = −165.2° for U1, χ = −116.8° for U2), with the ribose moieties extending outward from the pocket (Fig. 7 ▸ *b*). Interestingly, while the U1 ribose is in the 3′-*endo* conformation typically seen in canonical (A-form) RNA structures, with a pseudo-rotation phase angle (*P*) of 17.5° for this North sugar pucker, the U2 ribose adopts a less typical 2′-*endo* conformation (*P* = 163.2°).

**Figure 8:**
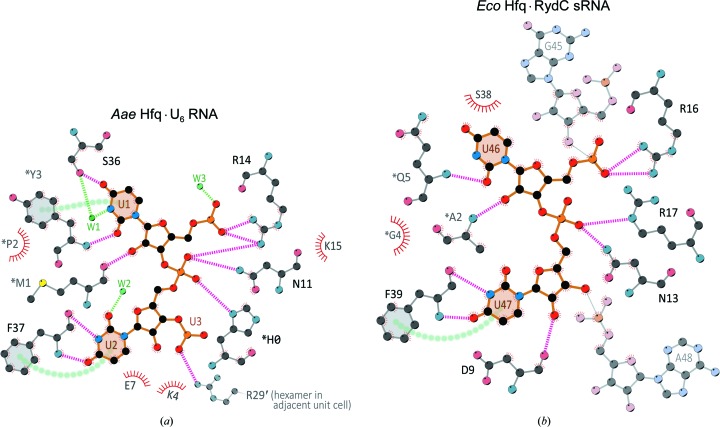
Conserved pattern of interatomic contacts at the lateral RNA-binding site of Hfq hexamers. In this schematic diagram of the interatomic contacts between the lateral site of *Aae* Hfq, U_6_ RNA and nearby H_2_O molecules (*a*), protein atoms are shown as ball-and-stick representations (CPK coloring, light gray C atoms) and covalent bonds in the nucleotides are drawn as thicker, orange-colored lines. For clarity, only a subset of H_2_O molecules is drawn (green, labeled ‘W#’). Here, asterisks denote another Hfq chain in the same unit cell and the prime symbol denotes a neighboring cell. Hydrogen bonds are magenta for protein⋯RNA interactions, while those to H_2_O are shown in green. Stacking interactions between the aromatic entities φ_1_ and φ_2_ are indicated by green circles from φ_1_⋯φ_2_. Two nucleotides of uridine (labeled) appear in an open, bridging conformation with the α-helix and β2 strand of an Hfq monomer (gray flanking regions). The phosphate groups are hydrogen-bonded to Asn11 and Arg14 of the N-terminal α-helix, while the nucleobase hydrogen bonds to the backbone atoms of strand β2 (specifically, Ser36 and Phe37), thus imparting specificity for uridine. Note that additional π-stacking interactions are present between the side chain of Phe37 and RNA base U2, as well as within the RNA (between U2⋯U1; not shown for clarity). The lateral pocket of *Eco* Hfq is shown in (*b*), complexed with the sRNA RydC [same coloring scheme and conventions as in (*a*)]. The U46 and U47 bases adopt conformations similar to those seen in (*a*), with the phosphate groups contacting residues of the α-helix. Phe39 π-stacks with U47, analogous to the interaction seen in *Aae* Hfq. Note that the adjacent G45 and A48 bases are flipped away from the pocket and are shown here to offer context in the overall sequence of the sRNA. While not strictly conserved in terms of precise amino-acid sequence, the N-terminal regions of the *Aae* and *Eco* Hfq homologs do provide similar backbone interactions with U1 and U46, respectively. Note also the directionality of the RNA backbone, which follows the same 5′→3′ path along the lateral site on the surface of the *Aae* and *Eco* Hfq rings (see also Figs. 7 ▸ *a* and 7 ▸ *b*).

Protein⋯RNA interactions are mediated by both side-chain and backbone atoms of *Aae* Hfq. The full set of interactions is shown in three dimensions in Figs. 7 ▸(*a*) and 7 ▸(*b*), and schematically in Fig. 8 ▸(*a*). Two side chains in the N-terminal α-helix of *Aae* Hfq, Asn11 and Arg14, contact the phosphodiester groups, and another cationic residue (Lys15) is 3.6 Å from the phosphodiester group linking the two uridines. Backbone and side-chain atoms from strand β2 hydrogen-bond to the bases, ensuring uridine specificity (Figs. 7 ▸ *b*, 8 ▸ and 9 ▸). In particular, both the carbonyl O atom and amide N atom of Phe37 interact with N3 and O4 of U2, respectively, while the hydroxyl side chain of Ser36 contacts the exocyclic O4 of the U1 nucleobase. Ser36 also helps position a pivotal H_2_O that directly hydrogen bonds to both the N3 atom of U1 and the Ser36 hydroxyl (Fig. 8 ▸ *a*); this well ordered (ice-like) water molecule engages in a network of hydrogen bonds in a distorted tetrahedral geometry (additional structural waters also contact the uracil and phosphodiester moieties, as shown in Fig. 8 ▸). Other interactions at the lateral site include a series of three π-stacking interactions (Fig. 8 ▸ *a*): between the phenyl ring of Phe37⋯U2, between the U1⋯U2 bases and between the phenolic ring of *Tyr3⋯U1. RNA binding at the lateral site is composite in nature, involving not just residues of strand β2 and helix α1 of one Hfq subunit, but also the N-terminal tail of an adjacent subunit in the ring. The irregularly structured N-terminal tail of one Hfq monomer extends into the neighboring lateral site, where the N-terminal sequence H^0^M^1^P^2^Y^3^K^4^ nearly ‘covers’ this rim site and supplies additional contacts with RNA. For instance, *Tyr3 engages in the π-stacking mentioned above, as well as a hydrogen bond between its amide N atom and the O2 of U1 (an interaction that does not select between uracil and cytidine). Also in this region, the backbone carbonyl O atom of *Met1 hydrogen-bonds to the ribose O2′ of U1, thus contributing to discrimination between RNA and DNA. Finally, we note that two contacts in this region may be spurious: (i) the *His0⋯phosphodiester group interaction, where residue *His0 is from the recombinant construct (not wild-type *Aae* Hfq; see the numbering in Supplementary Fig. S1), and (ii) the Arg29′⋯phosphodiester group interaction, which is a crystal lattice contact (the prime symbol on Arg29′ indicates an adjacent unit cell).

**Figure 9:**
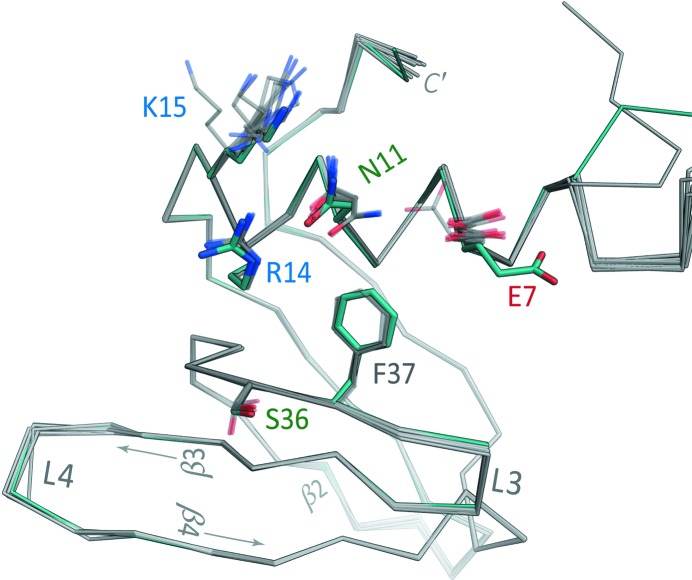
The lateral site of *Aae* Hfq is pre-structured for RNA binding. The three-dimensional structure of the single, unique monomer from the Hfq-U_6_ co-crystal structure (teal backbone) was superimposed with the 12 subunits of the apo Hfq structure (grey). Residues that contact RNA, to within ∼3.6 Å in the *P*6 Hfq·U_6_ structure, are shown as sticks for both the *P*6 and *P*1 structures. Apart from residue Glu7, which sterically occludes the binding pocket and thus is likely to adopt a different conformation upon RNA binding, note that the side chains in the apo structure adopt rotameric states quite similar to those in the three-dimensional structure of U_6_-bound *Aae* Hfq. This finding suggests pre-organization of the RNA-binding site of *Aae* Hfq.

Comparison of the *Aae* Hfq·U_6_ structure with the independently refined apo *Aae* Hfq structure suggests that the lateral RNA-binding site is essentially pre-structured for RNA complexation (Fig. 9 ▸). In terms of comparative structural analysis, note that the apo/*P*1 and RNA-bound/*P*6 structures (i) are at equally high resolutions (1.49 and 1.50 Å, respectively; Table 1 ▸), (ii) were refined in similar manners (*e.g.* using anisotropic ADPs), albeit independently of one another, and (iii) are of comparable quality in terms of *R*_work_/*R*_free_, stereochemical descriptors *etc.* (Table 2 ▸). Residues Asn11, Arg14, Ser36 and Phe37, which are phylogenetically conserved to varying degrees (Fig. 1 ▸), largely define the structural and chemical topography of the lateral site (Fig. 7 ▸ *a*). As shown in Fig. 9 ▸, these crucial residues adopt nearly identical rotameric states in the apo and U_6_-bound forms of *Aae* Hfq. The two principal RNA-related structural differences on going from the apo to the U_6_-bound forms are (i) a shift in the Glu7 rotamer (Fig. 9 ▸, red label), positioning this side chain away from the pocket and thus enabling the U2 base to be accommodated, and (ii) the precise path of the N-terminal tail (*i.e.* the ∼5 residues preceding helix α1), which varies with respect to the lateral site. In the dodecameric apo structure, six of the N-termini mediate ring⋯ring contacts (Fig. 5 ▸, DE ring) while the other half (from the PE ring) mediate lattice contacts, giving rise to one source of structural heterogeneity in this region. In terms of intrinsic conformational flexibility, normal-mode calculations (Supplementary Fig. S6 and §2.6) indicate that the N-terminal regions in the hexamer are highly flexible when free in solution, but rigidified (as much as any other part of the Sm fold) when sandwiched between the Hfq rings.

## 4. Discussion   

The apo form of *Aae* Hfq, refined to 1.49 Å resolution in space group *P*1, reveals a dodecamer comprised of two hexamers in a head-to-tail orientation. The individual subunits of *Aae* Hfq are similar in structure, with a mean pairwise r.m.s.d. of less than ∼0.3 Å for all monomer backbone atoms. The largest differences among the 13 independently refined Hfq monomer structures (12 in *P*1, one in *P*6) occur in the N-terminal and L4 loop regions; notably, these are the two regions that mediate much of the interface between rings (distal⋯proximal face contacts in Fig. 5 ▸), as well as the intermolecular contacts between dodecamers across the lattice. The patterns of structural differences are also captured in the symmetric matrix of pairwise r.m.s.d.s between chains: hierarchical clustering on this distance matrix results in the monomers that comprise the PE (chains *A*–*F*) and DE (chains *G*–*L*) hexameric rings partitioning into two distinct groups (Fig. 6 ▸ *c*, Supplementary Fig. S5*c*).

Sm proteins, including Hfq, exhibit a strong propensity to self-assemble into cyclic and higher-order oligomers. These assemblies often crystallize as either (i) cylindrical tubes with a defined polarity, *via* a head→tail association of rings (*Aae* Hfq and *Mth* SmAP1 are two examples) or (ii) head↔head stacks of cyclic oligomers, often with dihedral point-group symmetry (*Pae* SmAP1 is an example; Mura, Kozhukhovsky *et al.*, 2003 ▸). An examination of the lattice packing of all known Hfq structures (data not shown) reveals at least one example of each possible ring-stacking mode for a dodecameric assembly: (i) a proximal·proximal interface, as seen in the extensive interface between hexamers of an Hfq ortholog from the cyanobacterium *Synechocystis* sp. PCC6803 (PDB entry 3hfo; Bøggild *et al.*, 2009 ▸), (ii) a distal·distal interface, observed in *Staphylococcus aureus* Hfq (PDB entry 1kq2; Schumacher *et al.*, 2002 ▸) and in *P. aeruginosa* Hfq, with a more modest interface and relative translational shift of one ring (PDB entry 4mmk; Murina *et al.*, 2014 ▸) and (iii) the head→tail packing of two rings in the *Listeria monocytogenes* (*Lmo*) Hfq structure in apo and RNA-bound forms (PDB entry 4nl2; Kovach *et al.*, 2014 ▸). The *Aae* head-to-tail interface (Fig. 5 ▸) buries more ASA than that between the *Lmo* Hfq rings, but otherwise the stackings in these two Hfq structures resemble one another even in fine geometric detail (*e.g.* the top/bottom, PE/DE, rings are similarly rotated with respect to one another). Also, the *S. aureus* distal·distal dodecamer buries 2666 Å^2^ of surface area, which is considerably less than the ∼3700 Å^2^ of ΔSASA determined here for the distal·proximal stacking mode of *Aae* Hfq.

As a point of reference, note that the above ΔSASA quantities represent less buried surface area than in the ring–ring interfaces found in the structures of various Sm and SmAP homologs. (Recall that Hfq rings are hexameric while SmAPs are generally heptameric, meaning that a systematic difference in ΔSASA trends will occur simply by virtue of subunit stoichiometry.) The ring–ring interfaces in the *Pyro­baculum aerophilum* and *Methanobacterium thermautotro­phicum* 14-mers occlude 7550 and 3000 Å^2^, respectively. Unlike *P. aerophilum* SmAP3, where the burial of >21 000 Å^2^ along an intricate interface between stacked rings suggests *bona fide* higher-order oligomers (Mura, Phillips *et al.*, 2003 ▸), the extent of the *Aae* Hfq distal·proximal interface does not as clearly indicate whether or not dodecamers exist. The free energy of association betweens the PE and DE rings of *Aae* Hfq, Δ*G* ^o^_bind_, can be estimated *via* the linear relationship Δ*G* ^o^_bind_ = γBSA (the slope, γ, is often taken as ∼20–30 cal mol^−1^ Å^−2^; Janin *et al.*, 2008 ▸); however, the PE·DE interface of *Aae* Hfq is not primarily apolar in character, so this approach may severely overestimate the Δ*G* ^o^_bind_. Also, in terms of the existence and potential relevance of double rings and higher-order species, recall that *Aae* Hfq can form dodecamers *in vitro*, at least when bound to an A-rich RNA and assayed by AnSEC (Fig. 3 ▸ *b*, blue arrow). Nevertheless, despite all of these observations, (i) whether or not Hfq dodecamers actually occur *in vivo*, beyond crystalline and *in vitro* milieus (such as in AnSEC experiments) remains unclear, and (ii) even if such dodecamers do exist, the potential physiological activities and functional roles of higher-order oligomeric states of Hfq remain murky.

Intriguingly, our solution-state AnSEC data are consistent with the binding of A_18_, presumably at the distal face of (Hfq)_6_, causing a shift in the distribution of *Aae* Hfq oligomeric states from hexamers (only) to a more dodecameric population (Fig. 3 ▸). This effect may be attributed to the longer A_18_ strand simultaneously binding to two Hfq rings, giving a ‘bridged’ ternary complex. There also appears to be some length-dependence of the interaction of A-rich RNAs with Hfq, as we found that A_6_ did not exhibit high-affinity binding to *Aae* Hfq; this dependence may stem from mechanistic differences in the early (initiation) stages of the kinetic mechanism for Hfq⋯RNA binding. *Aae* Hfq demonstrates a nanomolar affinity for A_18_ and U_6_ RNA that is selective (C_6_ does not bind) and that is consistent with the properties of Hfq homologs characterized from other bacteria, both Gram-negative (*e.g.* proteobacteria such as *E. coli*) and Gram-positive. For instance, the magnesium-dependence of the *Aae* Hfq·U_6_ interaction (Fig. 4 ▸), with tenfold stronger binding in the presence of Mg^2+^, mirrors the Mg^2+^-dependency of U-rich binding by Hfq homologs from the pathogenic, Gram-positive bacterium *L. monocytogenes* (*Lmo*) and the Gram-negative *E. coli* (*Eco*; Kovach *et al.*, 2014 ▸). For both *Lmo* and *Eco* Hfq, the inclusion of 10 m*M* magnesium increased the U_6_-binding affinity by >100-fold; the effect was similar, but less pronounced, for U_16_ (an ∼3–4-fold increase). Thus, the Mg^2+^-dependency of the *Aae* Hfq·U_6_ RNA interaction is intermediate between these two extremes.

At present, only two other known Hfq structures contain a nucleic acid bound to the lateral site. These structures are (i) *Pae* Hfq co-crystallized with the nucleotide uridine 5′-tri­phos­phate (UTP; PDB entry 4jtx; Murina *et al.*, 2013 ▸) and (ii) *Eco* Hfq bound to a full-length sRNA known as RydC (PDB entry 4v2s; Dimastrogiovanni *et al.*, 2014 ▸). Comparison of the lateral RNA-binding sites of the *Aae, Pae* and *Eco* Hfq structures reveals a highly conserved pocket formed by Asn13, Arg16, Arg17, Ser38 and Phe39 (*Eco* Hfq numbering; see also Fig. 1 ▸). In *Aae* Hfq, Lys15 appears to be homologous to Arg16 in *Eco* Hfq, insofar as this side chain is well positioned to engage in electrostatic and hydrogen-bond interactions with the sugar-phosphate backbone of a bound RNA (Figs. 7 ▸ *b*, 8 ▸ and 9 ▸). This structural feature can be seen both in *Eco* Hfq (Arg17 with the phosphate of a neighboring nucleotide) and in *Pae* Hfq (Lys17 with the 5′-phosphate tail of UTP). Notably, uridine is the only nucleotide that has been found to bind at the lateral site in all three of these Hfq structures: *Eco* Hfq, *Pae* Hfq and now *Aae* Hfq.

At a resolution of 1.5 Å, the *Aae* Hfq·U_6_ structure offers new insights into the apparent specificity of the lateral pocket for uridine nucleosides. We see that interactions with the backbone of strand β2 provide discrimination between uracil and cytosine bases in the cognate RNA. One uracil base π-stacks with a key phenylalanine residue, while the second uracil stacks atop the preceding nucleobase. The second nucleotide adopts a C2′-*endo* conformation, leading to the accommodation of the base in this binding cleft on the surface of Hfq. In this configuration, the N-terminal region may then provide further enthalpically favorable interactions that stabilize the complex. The *Aae* Hfq lateral site includes two of the three arginine residues of the ‘arginine patch’ known to be important for annealing of sRNAs and mRNAs (Panja *et al.*, 2013 ▸). We propose that the third arginine of this motif acts primarily electrostatically (without directionality, and non­specifically as regards RNA sequence) in order to enhance the diffusional association of an RNA by ‘guiding’ it towards the lateral pocket. In addition, the physicochemical basis for the phylogenetic conservation of the lateral site may be that it simply provides additional surface area for Hfq⋯sRNA interactions, perhaps supplying an extended platform for the ‘cycling’ of RNAs across the surface of the Hfq ring (Wagner, 2013 ▸); similarly, the rim site may serve as an additional ‘anchor’ site for the association of moderate-length, U-rich RNAs that bind with low intrinsic affinity for the proximal site, but which can reach the lateral/rim site. We propose that the lateral site, which is structurally well defined on the outer rim of the *Aae* Hfq hexamer, is a biologically relevant region that functions in binding (U)*_n_* segments of RNA containing at least two consecutive uridine nucleotides; moreover, we propose that this RNA-binding region is conserved in even the most ancient bacterial lineages.

The structural features of Hfq⋯RNA interactions in homologs from evolutionarily ancient bacteria share some similarity with the properties of Sm-like archaeal proteins (SmAPs), such as a SmAP from the hyperthermophile *Pyrococcus abyssi* (*Pab*) that was co-crystallized with U_7_ RNA (Thore *et al.*, 2003 ▸). Interestingly, the oligoribonucleotide in that crystal structure was found in two sites: the canonical U-rich binding site near the lumen of the ring (analogous to the proximal site of Hfq), as well as a ‘secondary’ pocket on the same (proximal) face. This secondary site of *Pab* SmAP is distant from the U-binding site, lying between the N-terminal α-helix and strand β2 of the Sm fold. Note that the ‘lateral site’ of Hfq had not yet been discovered as an RNA-interaction region at the time of the *Pab* SmAP structure determination. The secondary RNA-binding site in *Pab* SmAP also contains a phenylalanine residue that is conserved among Hfq homologs and that is required for π-stacking with the nucleobase. However, the asparagine residue found at the lateral site of all characterized Hfq homologs is instead a histidine in *Pab* SmAP; the imidazole side chain of this residue provides an additional stacking platform for an adjacent ribonucleotide in the *Pab* complex, in an interaction that is not seen in known Hfq homologs. The α-helix of *Pab* SmAP does not extend as far as that of Hfq, and the arginine-rich patch that occurs at this rim area in Hfq homologs is but a single lysine residue in *Pab* SmAP. Nevertheless, the presence of this partially conserved lateral pocket in *Pab* SmAP does suggest an ancient, common origin for this mode of protein⋯RNA recognition by Hfq and other members of the Sm superfamily. Somewhat similarly, a uridine-binding site was crystallo­graphically identified in *Pyrobaculum aerophilum* SmAP1 in a region on the ‘L3 face’ (analogous to the proximal face of Hfq) that lies distal to the canonical U-rich RNA-binding site at the inner surface of the pore; this L3-face region was described as a ‘secondary’ binding site because of relatively weak electron density for the phosphoribose (Mura, Kozhukhovsky *et al.*, 2003 ▸). We can now see that the secondary U-rich binding sites in at least two archaeal Sm proteins, from *Pab* and *P. aerophilum*, occupy a region that is roughly analogous to the lateral rim of Hfq.

The historical lack of structural data on RNA binding at the Hfq lateral site may be because uridine-rich RNAs, such as might localize to the lateral rim, are also capable of binding to the higher-affinity proximal site. A single binding event is consistent with the idealized shape of our *Aae* Hfq·U_6_ binding curves (Fig. 4 ▸), which bear no hint of multiple transitions or non-two-state binding. This could indicate that U_6_ binding at the proximal and lateral sites differs by at least an order of magnitude (beyond the detection range of our assay). In terms of the structure of the *Aae* Hfq·U_6_ complex reported here, we suspect that two facets of our crystallization efforts serendipitously shifted the RNA-binding propensity towards the lateral site. Firstly, MPD was present at high concentrations in our crystallization condition (many Hfq homologs reported in the literature were crystallized with PEGs, not MPD). MPD is a commonly used precipitating agent and cryoprotectant, and inspection of electron-density maps reveals it to be associated, at high occupancy, with all 12 subunits of the apo form of *Aae* Hfq; specifically, 24 of the 25 MPDs found in the *P*1 electron density are bound in one of two locations (Fig. 5 ▸), and one of these locations corresponds to what would be a proximal RNA-binding site. Moreover, an MPD molecule was also bound in the *P*6 (U_6_-bound) crystal forms, in clear density at the proximal site (Fig. 7 ▸); notably, this proximal-site MPD almost perfectly superimposes in three dimensions with the 12 MPDs at this site in the 12 subunits of the apo/*P*1 structure. In terms of structural and chemical properties, the hydroxyl groups of MPD closely mimic the ribose and uracil moieties of uridine, as shown in Supplementary Fig. S8. Residues His56 and Gln8 have been identified as two key residues in the proximal site that contact the ribose 2′-OH and the exocyclic O2 atom of uracil upon binding of U_6_ at the proximal site (Schumacher *et al.*, 2002 ▸). However, in our *Aae* Hfq structure these two residues instead contact MPD (*Gln6 and His56 in Fig. 7 ▸ *c*). The lateral RNA-binding site, however, does not include many contacts to ribose (*versus* the phosphate and nucleobase groups) and thus MPD would not be expected to compete as strongly against RNA binding at that site. The hypothesis that MPD interferes with RNA binding by localizing at the proximal site (see, for example, Fig. 7 ▸ *c*) is borne out by RNA-binding competition assays, which reveal that exceedingly high concentrations of MPD, such as in our crystallization conditions, can successfully inhibit *Aae* Hfq·U_6_ binding (Supplementary Fig. S9). The second unique feature of *Aae* Hfq that may increase the affinity for U-rich RNA at the lateral site is the flexible N-terminal tail, which folds over the lateral site when nucleic acid is bound, further stabilizing the associated U_6_ RNA. In our work, the N-terminus includes three plasmid-derived residues that remain after the cleavage of the 6×His tag used in protein purification (G^−2^S^−1^H^0^; Supplementary Fig. S1*a*). The additional histidine contacts the phosphate of nucleotide U2 (Figs. 7 ▸ *b* and 8 ▸). In addition, the native sequence includes a tyrosine residue that provides further aromatic stacking interactions with base U1 (residue *Tyr3 in Figs. 7 ▸ *b* and 8 ▸). This tyrosine residue is not conserved among other Hfq homologs, many of which contain a glutamate at this position (Fig. 1 ▸).

The crystallographic and biochemical work reported here reveals that the putative Hfq homolog encoded in the *A. aeolicus* genome is an authentic Hfq, as it (i) adopts the Sm fold, (ii) self-assembles into hexameric rings that can associate into higher-order double rings in the lattice (as do many known Hfqs) and (iii) binds A/U-rich RNAs with high affinity (and selectivity). Perhaps most excitingly, these structural and functional properties are recapitulated by an Hfq homolog from the Aquificae phylum, which may be the most basal, deeply branching lineage in the bacterial domain of life (Bocchetta *et al.*, 2000 ▸; Burggraf *et al.*, 1992 ▸). To date, all Hfq structures have been limited to three phyla: (i) most Hfq structures are from the Proteobacteria, (ii) a few are from the (mostly Gram-positive) Firmicutes and, finally, (iii) two known homologs are of cyanobacterial origin. Because of its basal phylogenetic position, the *Aae* Hfq structures reported here, the first Hfq structures from outside these three bacterial lineages, suggest that members of the Sm/Hfq superfamily of RNA-associated proteins, along with at least some of their RNA-binding properties, are likely to have existed in the last common ancestor of the Bacteria.

## Supplementary Material

PDB reference: *A. aeolicus* Hfq dodecamer in space group *P*1, 5szd

PDB reference: *Aquifex aeolicus* Hfq bound to a U-rich RNA, 5sze

Supporting Information.. DOI: 10.1107/S2059798317000031/yt5100sup1.pdf

## Figures and Tables

**Table 1:** X-ray diffraction data-collection and processing statistics Values in parentheses are for the highest resolution shell.

	*Aae* Hfq, apo form (‘*P*1’)	*Aae* Hfq·U_6_ RNA (‘*P*6’)
Diffraction source	24-ID-E, APS NE-CAT	24-ID-C, APS NE-CAT
Wavelength (Å)	0.9792	0.9195
Temperature (K)	100	100
Detector	ADSC Q315 CCD	Dectris PILATUS 6MF
Crystal-to-detector distance (mm)	200	300
Rotation range per image (°)	1.0	1.0
Total rotation range (°)	400.0	300.0
Exposure time per image (s)	1.0	1.0
Space group	*P*1	*P*6
*a*, *b*, *c* (Å)	63.46, 66.06, 66.10	66.19, 66.19, 34.21
α, β, γ (°)	60.05, 83.94, 77.17	
Mosaicity (°)	0.143	0.107
Resolution range (Å)	57.27–1.49 (1.53–1.49)	34.21–1.50 (1.55–1.50)
Total No. of reflections	299450	46203
No. of unique reflections	138120	13177
Completeness (%)	93.7 (83.7)	94.9 (93.4)
Multiplicity	2.2 (2.1)	3.5 (3.5)
〈*I*/σ(*I*)〉	14.0 (3.4)	12.3 (3.6)
*R*_merge_ †	0.039 (0.258)	0.056 (0.292)
*R*_meas_ ‡	0.052 (0.349)	0.065 (0.345)
*R*_p.i.m._ ‡	0.035 (0.234)	0.032 (0.179)
CC_1/2_ §	0.998 (0.886)	0.998 (0.942)
Overall *B* value from Wilson plot (Å^2^)	12.62	15.87
Matthews coefficient *V*_M_ (Å^3^ Da^–1^)	2.06 [12 subunits in asymmetric unit]	2.28 [one subunit in asymmetric unit]
Solvent content (%)	40.21	46.08

† *R*_merge_ = 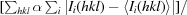 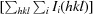, where *I_i_*(*hkl*) is the intensity of the *i*th observation of reflection *hkl*, 〈.〉 denotes the mean of symmetry-related (or Friedel-related) reflections and the coefficient α = 1; the outer summations run over only unique *hkl* with multiplicities greater than one.

‡ *R*_meas_ is defined analogously to *R*_merge_, save that the prefactor α = [*N_hkl_*/(*N_hkl_* − 1)]^1/2^ is used; *N_hkl_* is the number of observations of reflection *hkl* (index *i* = 1→*N_hkl_*). Similarly, the precision-indicating merging *R* factor, *R*_p.i.m._, is defined as above but with the prefactor α = [1/(*N_hkl_* − 1)]^1/2^.

§ CC_1/2_ is the correlation coefficient between intensities chosen from random halves of the full data set.

**Table 2:** Structure determination and model refinement Values in parentheses are for the highest resolution shell.

	*Aae* Hfq, apo form (‘*P*1’)	*Aae* Hfq·U_6_ RNA (‘*P*6’)
Resolution range (Å)	46.35–1.49 (1.51–1.49)	34.21–1.50 (1.56–1.50)
Completeness (%)	93.9	94.9
No. of reflections, working set	138104 (12739)	13171 (1308)
No. of reflections, test set	10625 (983)	662 (70)
Final *R*_cryst_	0.1323 (0.1531)	0.1443 (0.1499)
Final *R*_free_	0.1696 (0.2108)	0.1719 (0.1933)
No. of non-H atoms		
Macromolecules	7670 Hfq	598 Hfq, 43 RNA
Ligands	200 MPD, 32 Gnd, 7 Cl^−^, 28 PEG	8 MPD, 7 PEG
Solvent	413 H_2_O	36 H_2_O
Total	8350	692
No. residues of protein, solvent or ligand molecules included in the final, refined structure		
*Aae* Hfq	848 [over 12 subunits]	71 [over 1 subunit]
H_2_O	413	36
U_6_ RNA		∼2–3†
MPD	25	1
Cl^−^	7	
Gnd	8	
PEG‡	4	1
R.m.s. deviations		
Bonds (Å)	0.005	0.005
Angles (°)	0.75	0.76
Average *B* factors (Å^2^)		
Protein	19.32	22.18
Ligand	25.89	30.44
Ramachandran plot		
Most favored (%)	98	97
Allowed (%)	1.7	2.9
Outliers (%)	0	0
Rotamer outliers (%)	0.34	1.5
PDB code	5szd	5sze

† This value is given as a range because two complete U nucleotides, plus a fragment of a third residue, could be built into the electron-density maps.

‡ Fragments of polyethylene glycol could be built in both structures, generally of two to three repeat units [*i.e.* (O–C–C)_2_–O, neglecting H atoms].
