# Supplementary material for: Crystal structure and RNA-binding properties of an Hfq homolog from the deep-branching Aquificae: conservation of the lateral RNA-binding mode
Source: Acta Crystallogr D Struct Biol. 2017 Mar 31;73(Pt 4):294–315. doi: 10.1107/S2059798317000031 (PMC5379935; doi:10.1107/S2059798317000031)

STRUCTURAL
BIOLOGY

Volume 73 (2017)

Supporting information for article:

Crystal structure and RNA-binding properties of an Hfq homolog from the deep-branching Aquificae: conservation of the lateral RNA-binding mode

Kimberly A. Stanek, Jennifer Patterson-West, Peter S. Randolph and Cameron Mura

Supporting Information

Crystal Structure and RNA-binding Properties of an Hfq Homolog from the Deep-branching Aquificae: Conservation of the Lateral RNA-binding Mode

Kimberly A Stanek, Jennifer Patterson-West, Peter S Randolph, Cameron Mura*

Department of Chemistry; University of Virginia; Charlottesville, VA 22904-4319 USA; *cmura@muralab.org

Contents

This Supporting Information contains the following nine Figures, accompanying captions, and two Tables:

Figure S1: The full-length, His6×–tagged *Aae* Hfq recombinant construct

Figure S2: Colorimetric assays of the nucleic acid populations that co-purify with *Aae* Hfq

Figure S3: SEC-MALS analysis of the oligomeric states of Hfq in complex with U-rich and A-rich RNAs

Figure S4: Numerical fitting of Hfq•RNA binding data with a single-site, receptor-depletion model

Figure S5: Large-scale (dodecamer-level) and small-scale (monomer-level) structural variation in the *P*1 (*apo*) and *P*6 (RNA-bound) crystal structures

Figure S6: Conformational heterogeneity in the *P*1 and *P*6 forms: Anisotropic atomic displacement parameters (ADPs), and normal mode analysis of a coarse-grained model

Figure S7: Difference electron density maps for the *Aae* Hfq•U_6 dataset solved in either *P*6 or *P*1

Figure S8: Chemical and geometric similarity of MPD to uridine, and fragments thereof

Figure S9: MPD can inhibit U-rich RNA binding to Hfq

Table S1: Cloning and expression of the His6×–tagged *Aae* Hfq recombinant construct (primers, etc.)

Table S2: Crystallization conditions for *Aae* Hfq in the *P*1 and *P*6 forms

Supporting Figure Legends

Figure S1. The full-length, His6×–tagged *Aae* Hfq recombinant construct, from cloning to X-ray diffraction. The *Aae hfq* gene was cloned into a pET-28b(+) expression plasmid, yielding a His6×-tagged, full-length *Aae* Hfq construct with the amino acid sequence shown here (**a**). Bold residues are the 80-AA–long native (*wild-type*) *Aae* sequence; the first 20 AAs of the 100-AA construct are from the expression vector, which supplies a His_6 tag (blue) and a thrombin recognition site (red). The cut-site, verified by MALDI-TOF MS, is marked by an arrow; as indicated, the tripeptide $^{N'}G^{-2}S^{-1}H^{0}$ remains prepended to the native *Aae* sequence after proteolytic removal of the affinity tag. Recombinant *Aae* Hfq was readily over-expressed in *E. coli* and purified, as illustrated by the SDS-PAGE gel in (**b**). In this sample, lane 1 is the molecular weight marker; lanes 2/3 are pre–/post–induction cell lysates; lanes 4/5 are from the supernatant/pellet of the final (production) step of cell lysis; lanes 6/7 are the supernatant/pellet from the 75 °C heat-treatment step (see Methods); lane 8 is flow-through/eluate from the immobilized Ni^{2+}–affinity chromatography; and lanes 9 and 10 are two elution fractions from the chromatographic step. Bands corresponding to the MW of monomeric and hexameric Hfq species are indicated by yellow schematics near lane 9. Buffers in the affinity chromatography steps included 6 M GndHCl in order to strip away contaminating nucleic acid (see Fig S2, below) and maintain *Aae* Hfq solubility, and this led to unavoidably severe smearing in these gel lanes (as described in the Methods section, GndHCl was removed in a later dialysis step). Sample specimens of *Aae* Hfq crystals, photographed under cross-polarized light (≈20 µm/edge; scale bar not shown), exhibit birefringence (**c**). The crystals are well-faceted and form in several different habits (including hexagonal plates), varying in size from ≈10-100 µm. The crystals yield high-quality X-ray diffraction patterns, such as the sample shown in (**d**).

Figure S2. Colorimetric assays of the nucleic acid populations that co-purify with *Aae* Hfq. When the denaturant GndHCl (see above) was not added to the purification workflow, a population of nucleic acids was persistently found to co-purify with *Aae* Hfq, as initially detected by the ratio of absorbance at 260 nm to that at 280 nm (A_{260}/A_{280}) exceeding ≈1.0. A systematic series of colorimetric assays (see Methods) helped identify the co-purifying nucleic acid as RNA (**a**). Briefly, the Benedict's reagent produces a color shift from blue to orange in the presence of free reducing sugars, the Bial's orcinol assay yields a green-blue product in the presence of a pentose sugar (such as ribose), and the Dische's diphenylamine test is specific for 2′-deoxyribose, yielding a blue product in the presence of DNA. Solutions containing 1 mg/mL ribose, 1 mg/mL RNA, or 3 mg/mL DNA were used as positive controls, and results are shown from a panel of positive and negative controls for each type of colorimetric reaction; water is also included as a generic negative control. The *Aae* Hfq–associated sample is shown in the fifth column, where a positive result can be seen with the Bial's assay but not the Benedict's or Dische's assays. The Hfq–co-purifying RNAs were separated via Trizol extraction and run on a 2% w/v agarose gel in order to assess their size distribution (**b**; lane 2). As seen by comparing to the molecular marker (lane 1), the main components of this RNA population are ≈100–200 nucleotides in length.

Figure S3. SEC-MALS analysis of the oligomeric states of Hfq in complex with U-rich and A-rich RNAs. To determine the molecular weights of different Hfq oligomeric states, alone and as various Hfq•RNA complexes, samples were analysed via SEC fractionation followed by multi-angle static light scattering (MALS) and refractive index measurements. SEC elution profiles (solid traces) were monitored via absorbance at 280 nm, and open circles (matching colors) give the molar mass distribution data—i.e., the computed mass (in kDa) versus elution volume. The weight-averaged molecular weight, M_w, of an Hfq sample is computed for the entire peak from this distribution, and the scale is given by the vertical axis on the right-hand side (colored axis). Panel (**a**) is essentially reproduced from the main text (Fig 3c) to aid comparisons with (**b**) and (**c**). The apparent M_w in (**a**), 58.75 kDa, corresponds to a hexameric assembly of *Aae* Hfq. The Hfq + U_6 sample (**b**) features a major peak with a M_w of 60.29 kDa, indicating a hexameric Hfq, presumably as part of an $(Hfq)_6$•RNA complex. A significant shift in the principal peak is found for the Hfq•A_{18} complex in (**c**). This new, shifted peak corresponds closely to an $(Hfq)_{12}$ assembly, with an apparent M_w of 119.30 kDa versus an ideal M_w of 119.87 kDa for a putative $\{(Hfq)_6\}_2$•A_{18} complex (113.79 kDa for 12 Hfq subunits + 6.08 kDa for A_{18} ssRNA).

Figure S4. Numerical fitting of Hfq•RNA binding data with a single-site, receptor-depletion model. The data plotted here are the same binding isotherms presented in Fig 4 of the main text, save that the abscissa is in terms of $[(Hfq)_6]$ rather than logarithmic units. FP assays were carried out using 5 nM FAM-U_6 (red) or FAM-A_{18} (blue) and varying concentrations of Hfq, either in the absence (thin lines) or presence (thick lines) of 10 mM $MgCl_2$. For each binding reaction, data from three replicates (standard errors given by vertical bars) were fit using the full, quadratic formula for the binding equation (§2.4, Eq 2); this model relaxes the assumptions that $[L]_{tot} \approx [L]$ and $[R]_{tot} \approx [R]$, thereby accounting for the phenomenon of receptor depletion at values near the K_D. When using this model to account for depletion of free *Aae* Hfq (treated as the receptor), note that the calculated binding constants (shown in the inset) will be systematically lower than those determined by any model that does not account for receptor depletion (e.g., Fig 4 of the main text). A caveat, however, is that this receptor–depletion model does not account for the possibility of cooperative interactions for binding at multiple sites. A hallmark of positive cooperativity is a sigmoidal binding curve (on the linear/linear, versus semi-log plot shown here) and, indeed, such was found to be the case for the binding of U-rich and A-rich RNAs to *Aae* Hfq.

Figure S5. The slight tilt between rings (δ) is attributable to differences in the *N*′-terminal regions, and the monomer structures partition into two clusters corresponding to the *PE* and *DE* hexamer rings. Panels (**a**) and (**b**) offer a structural analysis of the rigid-body rotations relating the two rings in the dodecamer of the *P*1 crystal form. Specifically, the two rings of the head–tail dodecamer—the proximal-exposed (*PE*) and distal-exposed (*DE*) hexamers—were brought, via pure rigid-body translation (no rotation), to a common origin (blue sphere in panels (**a**) and (**b**)), as described in Fig 6 of the main text. As labeled in panels (**a**) and (**b**), this difference essentially vanishes (**b**) when the *N*′-terminal regions are excluded from the calculation. At the level of individual monomer 3D structures, note that a total of 13 independently-refined *Aae* Hfq structures are

reported here (12 in *P*1 and one in *P*6). These 3D structures were compared by agglomerative hierarchical clustering on the distance matrix constructed from pairwise RMSDs. In the resulting dendrogram (**c**), each subunit in the dodecamer is labeled by its chain identifier (A, B, …, L), and two thumbnail schematics (inset) show the layout of the twelve chains in the two rings in the *P*1 cell (again, the *PE* ring is in cyan while the *DE* ring is in orange). The monomer of the *P*6 asymmetric unit (red) was also included in the clustering analysis, and it can be seen to cluster more closely with the *DE* ring (though only just barely, as it branches relatively near to the root of the tree). The RMSD scale of the distance matrix is indicated at the left. Note, in particular, two points: *(i)* The Hfq subunits within a ring are more similar in 3D structure to one another than they are to subunits in the other ring—i.e., *inter-ring* structural variation exceeds *intra-ring* variation. *(ii)* The circular clustering diagram in the main text (Fig 5c) is essentially a pruned representation of the results shown in (**c**); specifically, the circular graph can be constructed from the two deepest levels of this tree (the leaves, and one level shallower than the leaves).

Figure S6. Patterns of conformational heterogeneity in the *P*1 (*apo*; dodecamer) and *P*6 (U_6–bound; monomer) crystal forms: Anisotropic ADPs, and normal modes of a coarse-grained model. Three sets of panels are shown for *(i)* the *Aae* Hfq dodecamer refined in the *P*1 cell (panels (**a**), (**c**), (**e**)), and *(ii)* the *Aae* Hfq monomer in the *P*6 cell (panels (**b**), (**d**), (**f**)). Within each of the dodecamer panels, two perpendicular perspectives are supplied; arrows denote the relative orientation of these views. Panels (**a**) and (**b**) show 'putty' cartoon representations, with the diameter of the tubular backbone spline scaled by the magnitude of the *B*-factor field; because full, anisotropic ADPs were refined for most of the atoms in both *Aae* Hfq structures, the backbone scaling factors are not true B_{iso}, but rather B_{eq}, values computed from the full (anisotropic) B tensors (see *Methods*). In addition to scaling the tube diameter, the per-residue color in panels (**a**)→(**d**) is graded from blue (low B_{eq} values) to white (medium) to red (high). For clarity, the residues in a single subunit of the dodecamer are colored not on the blue→red grade, but rather from yellow→green (N′→C′ terminus) in panels (**a**) and (**c**). Also for clarity, a select few structural landmarks are denoted in panels (**a**)→(**d**), such as a few of the termini and L4 loops. Note that most of the elevated B_{eq} values occur at the termini (an unsurprising finding) as well as in loop L4; interestingly, this observation holds for both the dodecamer (**a**) and monomer (**b**). Panels (**c**) and (**d**) show the full, anisotropic ADPs for each atom, represented as thermal ellipsoids at the 50% probability level. Residues K51 and Q52, which are labelled in panel (**d**), lie within loop L4 and have particularly anisotropic ADPs in both the monomer and dodecamer structures; as a further example of this region's structural heterogeneity, alternate conformers were found to be useful in modelling the Q52 sidechain (see also Fig 6b of the main text). Using an anisotropic network model of inter-residue contacts, as described in the Methods section of the main text, normal modes were computed for a coarse-grained (C_α–only) representation of the *Aae* Hfq dodecamer and monomer. Panels (**e**) and (**f**) show displacement arrows to indicate projections of the structures along either the third non-trivial mode of the dodecamer (**e**) or the first three non-trivial modes (#7, 8, 9) of the monomer (**f**); these first three modes are colored red, orange, and yellow for the monomer. For the dodecamer, displacement arrows are shown along mode #3 for only half of the *DE* hexamer (red, bottom ring), and the opposite half of the *PE* hexamer (blue, top ring); this is done purely for the sake of clarity, and it clearly indicates the opposite rotational direction of the rings for this collective mode. Also for clarity, the extremely long displacement vectors are omitted from the first ≈3–4 *N*-terminal residues.

Figure S7. Difference electron density maps for the *Aae* Hfq•U_6 dataset solved in either *P*6 or *P*1. In separate workflows, diffraction datasets from the *Aae* Hfq•U_6 co-crystals were processed in either (**a**) *P*6 or (**b**) *P*1. Difference electron density maps (mF_o–DF_c), shown here contoured at 3.0σ, were computed after a single round of refinement of coordinates, occupancies and individual *B*-factors in PHENIX, using the general methodological approach described in the main text. Electron density for two complete nucleotides of uridine, along with a fragment of a third nucleotide, could be readily identified along the outer rim region of the Hfq ring (labelled green mesh). Notably, similar electron density was found at each of the six unique positions when the structure was solved in *P*1; the resulting pattern of electron density is comparable to that observed in the independent *P*6 solution.

Figure S8. Chemical and geometric similarity of MPD to uridine, and fragments thereof: A case of small-molecule mimicry in the proximal RNA-binding site of Hfq? Several distinct structural modes highlight the geometric and chemical similarity between MPD (2-methyl-2,4-pentanediol) and either (**a**) the uridine nucleo-

side (two configurations are shown), or (**b**) ribose alone, in the three unique configurations labelled here. This comparison was spurred by our observation that MPD—a frequently used cryo-protectant and precipitating agent in macromolecular crystallography—was found in precisely the same region of the *Aae* Hfq ring (namely, the proximal site) as might be expected for a U-rich ssRNA, such as the U_6 used in our co-crystallization efforts. Moreover, each MPD molecule engaged in the same chemical and geometric pattern of interactions found in other Hfq•U-rich RNA co-crystal structures (in which the U-rich RNA *is* bound in the proximal pore site). Specifically, we see in *Aae* Hfq•U_6 that the two hydroxyl moieties of MPD hydrogen-bond to the sidechains of *Q6 and H56 (Fig 7c), recapitulating the interactions between Q8 and H57 in the structure of *E. coli* Hfq bound to U_6 RNA (PDB ID 4PNO); these *Aae* Hfq···MPD interactions are noted in the leftmost model in panel (**a**). MPD also can be overlaid/matched to ribose alone, as illustrated in the three unique configurations of panel (**b**).

Figure S9. **MPD inhibits U-rich RNA binding to Hfq**. Competitive binding assays were performed via fluorescence polarization measurements of samples that contained Hfq, U_6 RNA, and various concentrations of MPD. Data from two series of assays are shown here, at 1 M (blue) and 400 mM (red) starting concentrations of MPD; in both series of assays, the MPD was serially-diluted into binding reactions containing 1 µM Hfq and 5 nM FAM-U_6. At sufficiently high concentrations of MPD, this small molecule can be seen to inhibit the binding of U_6 to Hfq, as indicated by a decreased polarization signal. An exact inhibition constant (K_i) could not be determined from these data, for various technical reasons––note that a clean low-FP asymptote is not reached at high [MPD] (even at >100 mM), and that complications arise from various countervailing effects. For instance, high solution viscosity at high [MPD] concentrations reduces molecular tumbling rates, thereby elevating the fluorescence anisotropy value for entirely spurious reasons (unrelated to RNA-binding); however, this effect directly opposes the increased fraction of freely tumbling FAM-U_6 upon inhibition of Hfq•U_6 binding. Though curve-fitting could not be performed, schematic lines are included as a visual guide to the upper (blue and red) and lower (blue, only) asymptotes. Note that concentrations of MPD beyond ≈ 100 mM, such as are often used in crystallization trials, can be seen to inhibit U_6···Hfq interactions. As a point of reference, the 35% v/v MPD used in our crystallization experiments (Supp Table S2) corresponds to an [MPD] ≈ 2.7 M.

Supporting Tables

Table S1. Cloning and expression of the full-length, His6×–tagged *Aae* Hfq recombinant construct.

Source organism	*Aquifex aeolicus* strain VF5
DNA source	*Aae* complete genome
PIPE forward primer (*insert*)	5′GCGCGGCAGCCA↓TATGCCTTACAAGTTGCAGGAGAGCTTTC3′
PIPE reverse primer (*insert*)	5′GTGGTGC↓TCGAGTTAACCTTGCCCCGGCACTCCTGCTTCTTC3′
PIPE forward primer (*vector*)	5′C↓TCGAGCACCACCACCACCACCACTGAGATCCGGCTGCTAAC3′
PIPE reverse primer (*vector*)	5′CA↓TATGGCTGCCGCGCGGCACCAGGCCGCTGCTGTGATGATGATG3′
Cloning vector	pET-28b(+)
Expression vector	pET-28b(+)
Expression host	*Escherichia coli* BL21(DE3)
Complete amino acid sequence of the recombinant construct that was produced	See Supp Fig S1

The *Nde*I (CATATG) and *Xho*I (CTCGAG) restriction sites are underlined, and arrows indicate the precise endonucleolytic cut-sites.

Table S2. Crystallization conditions for *Aae* Hfq in the *P*1 and *P*6 forms.

Method	Sitting-drop vapour diffusion
Plate type	VDX plates
Temperature (K)	291
Protein concentration	4.0 mg/ml
Buffer composition of protein solution	50 mM Tris pH 8.0; 500 mM NaCl
Composition of reservoir solution	0.1 M Sodium cacodylate; 5% (w/v) PEG 8000; 35% (v/v) MPD
Composition of additive	0.1 M Hexammine cobalt(III) chloride (*P*1 form)<br>1.0 M Guanidium HCl (*P*6 form)
Volume and ratio of drop	6 µl (3 µl protein + 2.4 µl reservoir + 0.6 µl additive)
Volume of reservoir	600 µl

Figure S1: The full-length, His6x–tagged *Aae* Hfq recombinant construct, from cloning to X-ray diffraction

(*a*)

```
                      thrombin cut site ↓0          10         20         30
MGSSHHHHHH SSGLVPRGSH MPYKLQESFL NTARKKRVKV SVYLVNGVRL
         40         50         60         70         80
QGRIRSFDLF TILLEDGKQQ TLVYKHAITT IVPHERLEIE FEEAGVPGQG
```

(*b*)

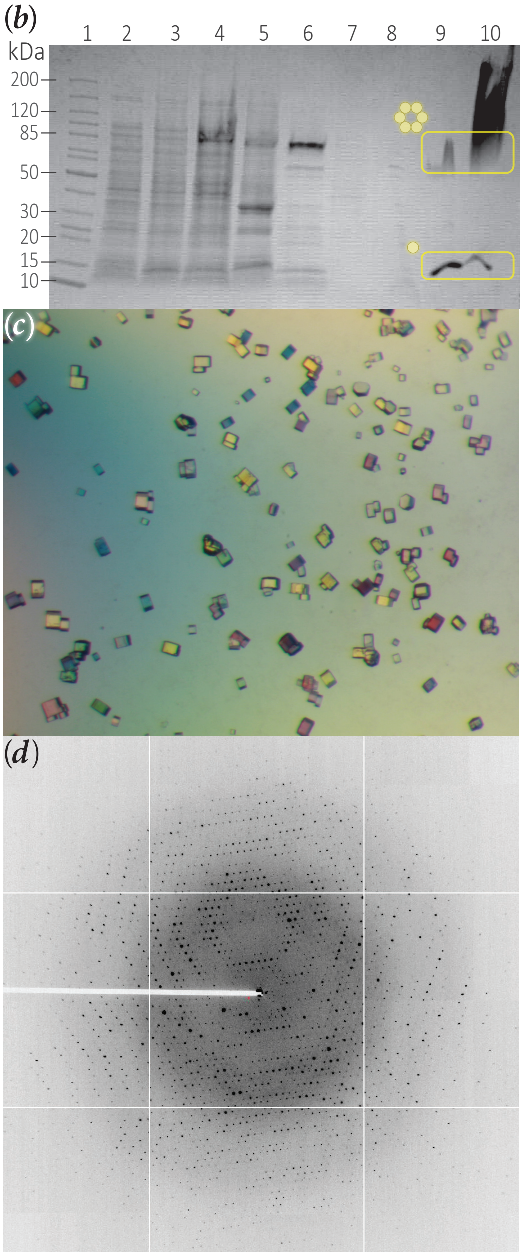

Figure S2: Colorimetric assays of the nucleic acid populations that co-purify with *Aae* Hfq

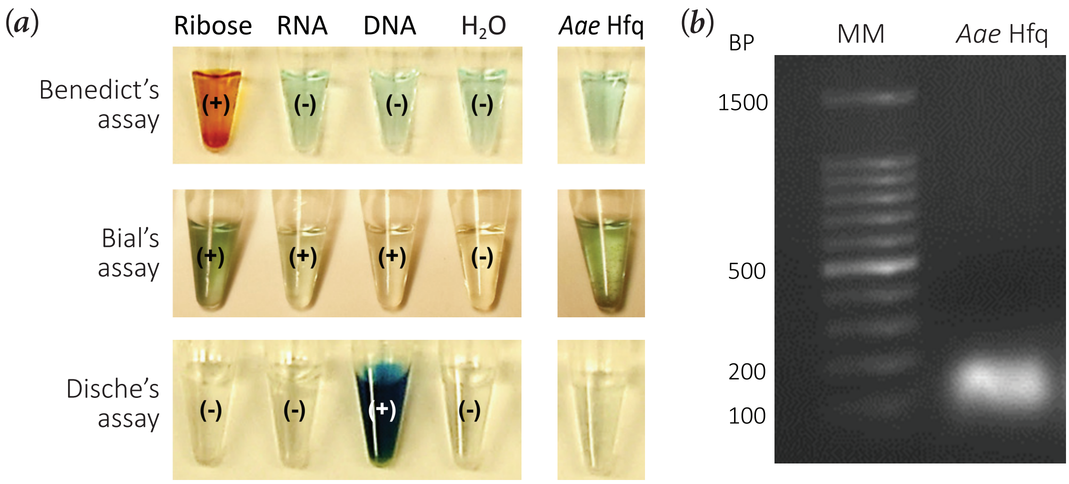

Figure S3: SEC-MALS analysis of the oligomeric states of Hfq in complex with U-rich and A-rich RNAs

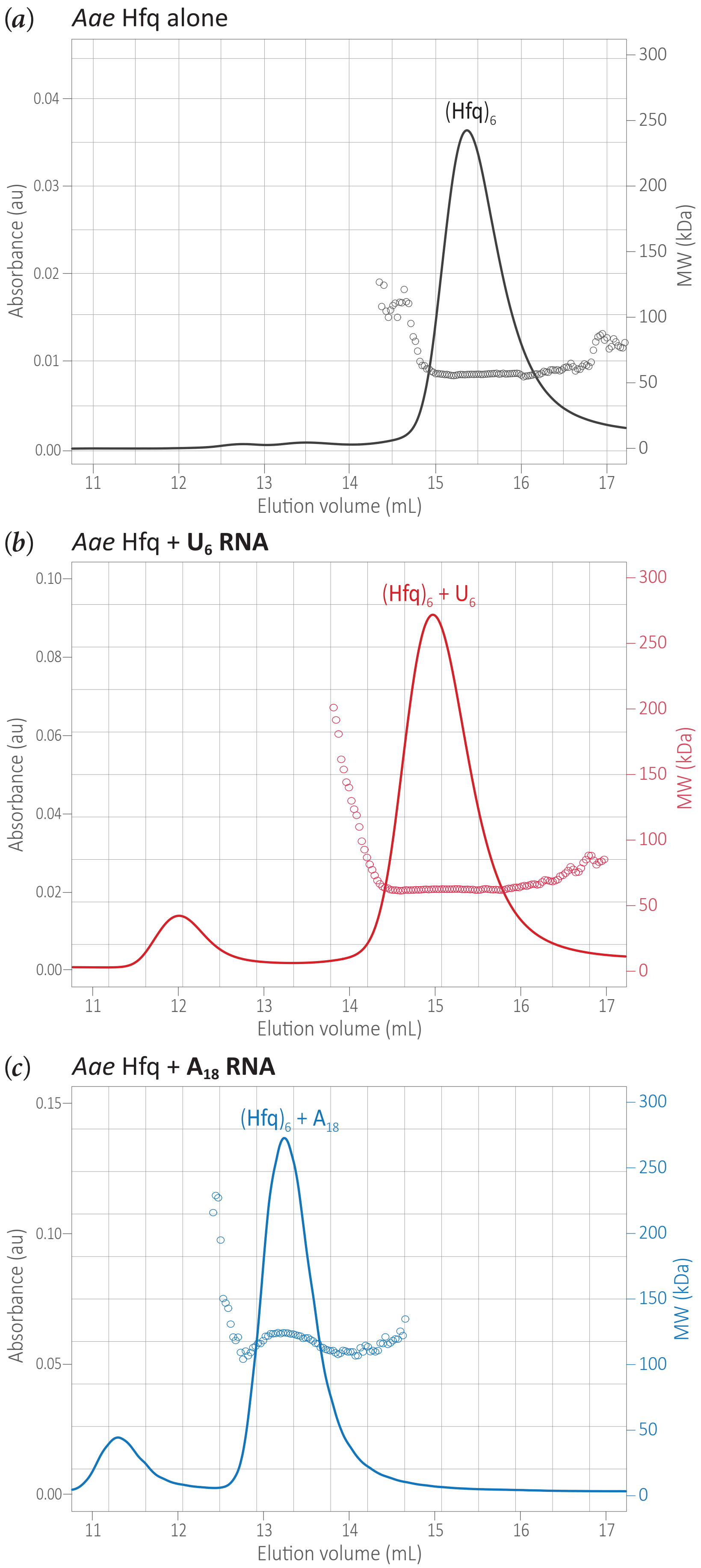

Figure S4: Numerical fitting of Hfq•RNA binding data with a single-site, receptor-depletion model

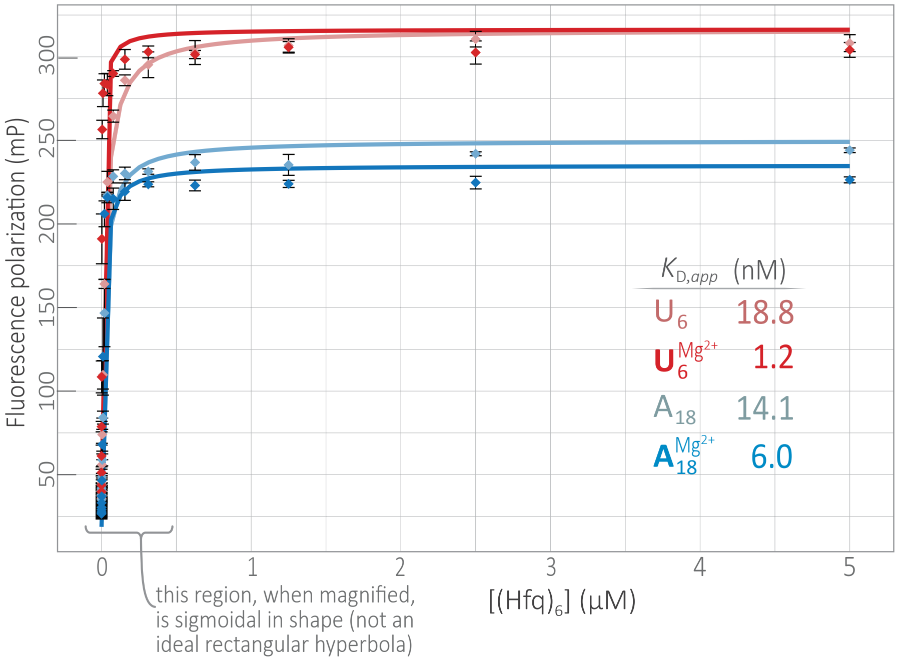

Figure S5: The slight tilt between rings (δ) is attributable to differences in the *N*′-terminal regions, and the monomer structures partition into two clusters corresponding to the *PE* and *DE* hexamer rings

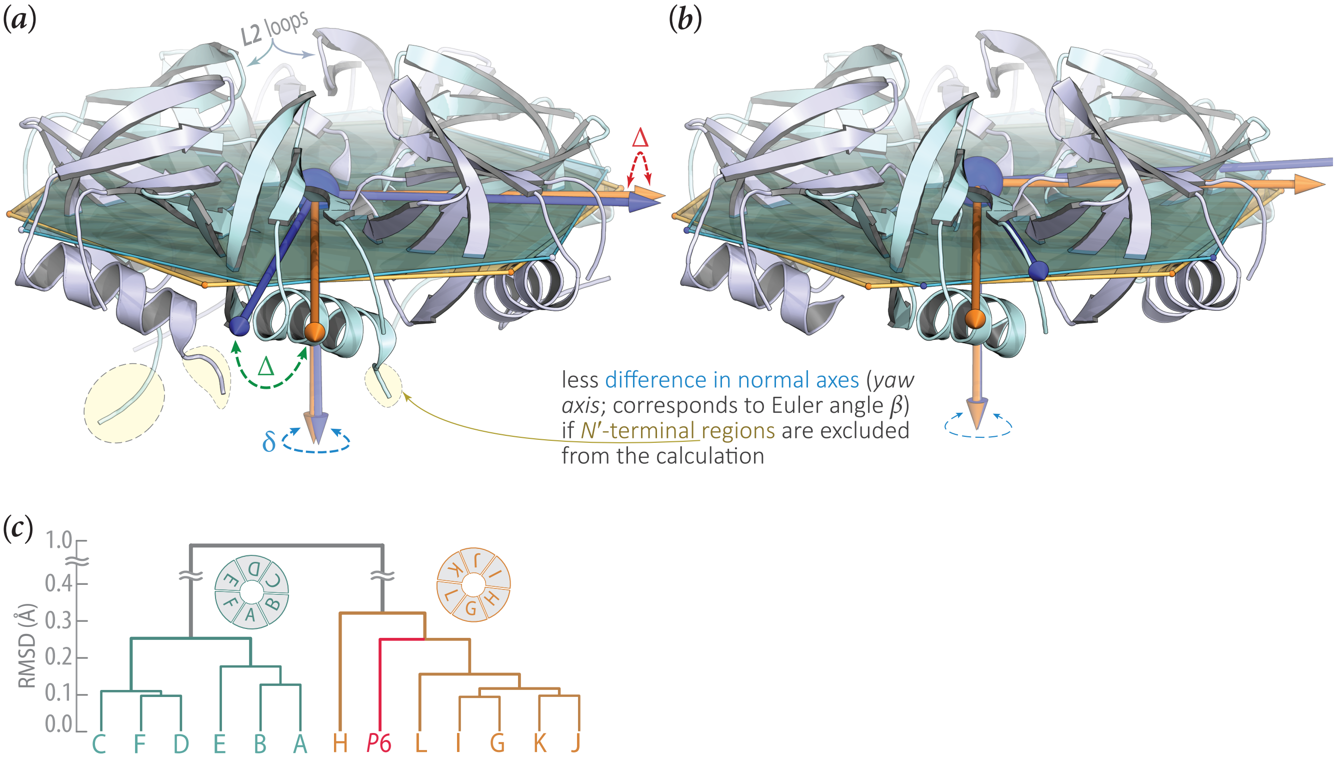

Figure S6: Patterns of conformational heterogeneity in the *P*1 (*apo*; dodecamer) and *P*6 (U_6–bound; monomer) crystal forms: Anisotropic ADPs, and normal modes of a coarse-grained model

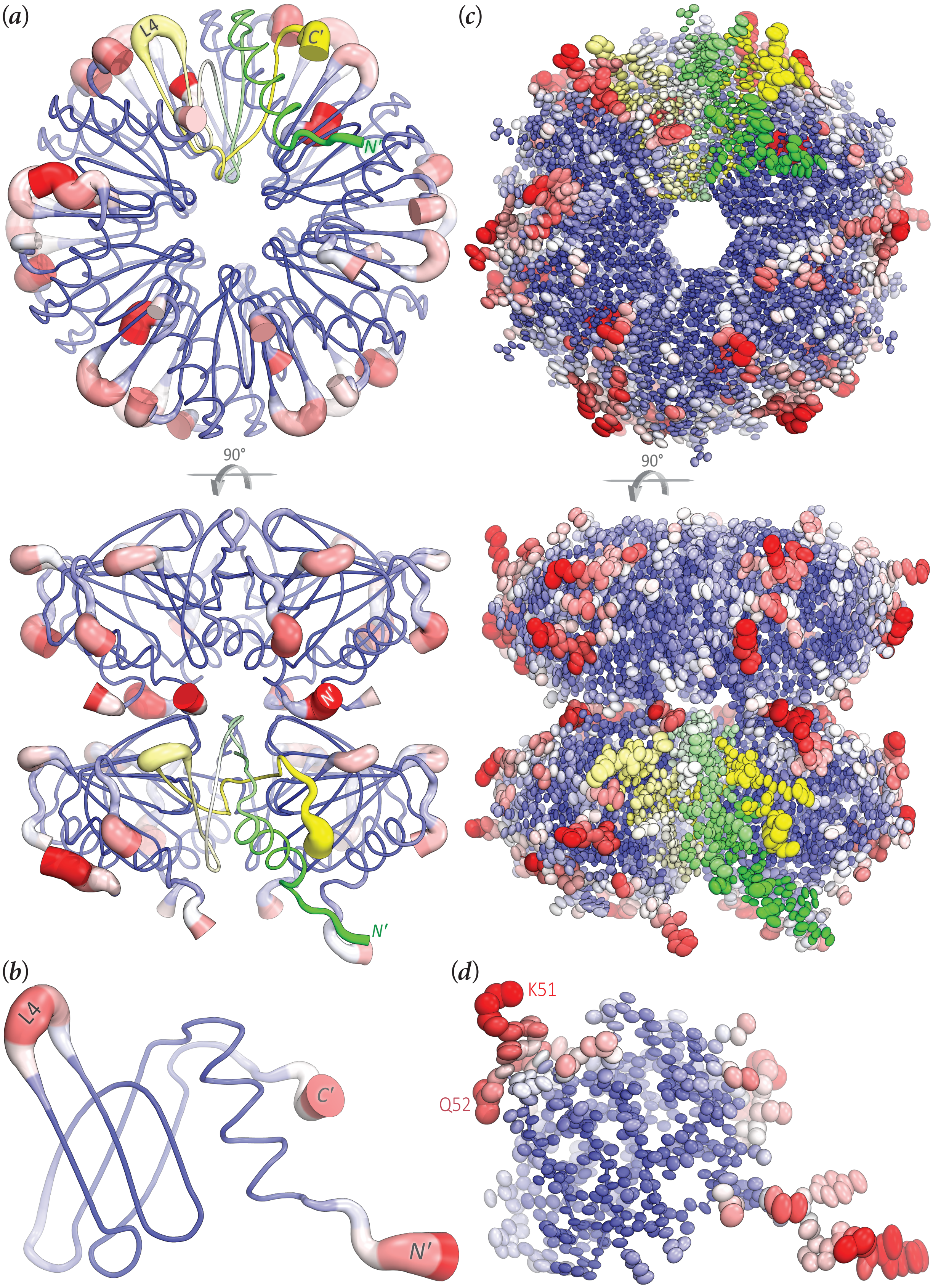

Figure S6: Patterns of conformational heterogeneity in the *P*1 (*apo*; dodecamer) and *P*6 (U_6–bound; monomer) crystal forms: Anisotropic ADPs, and normal modes of a coarse-grained model

(*e*)

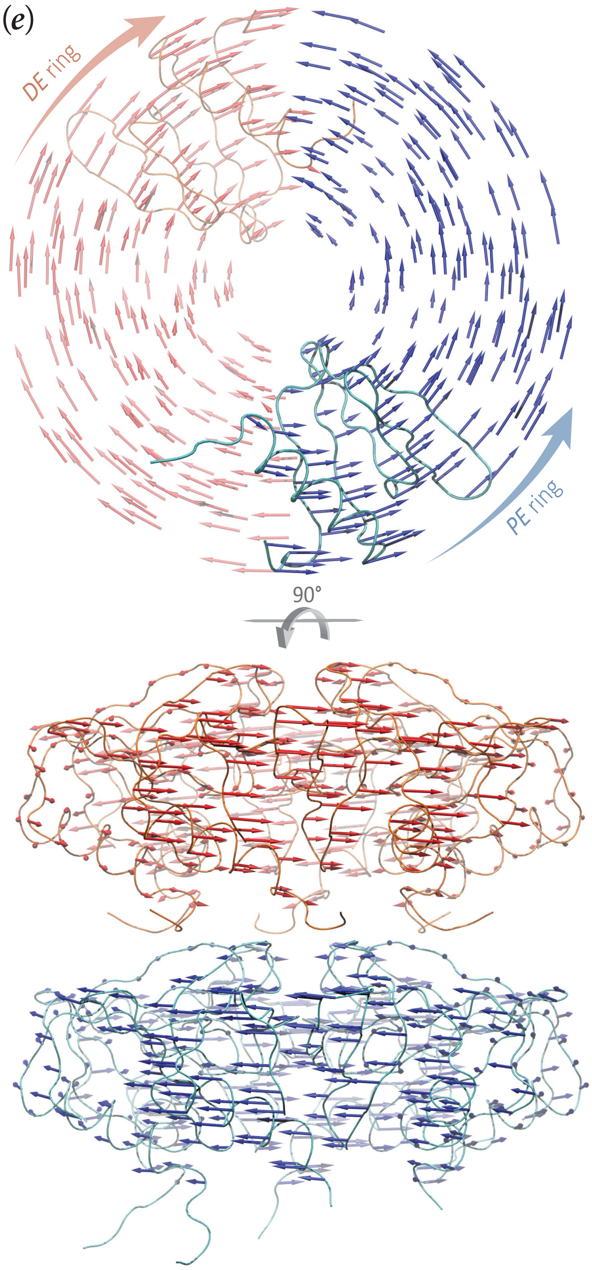

(*f*)

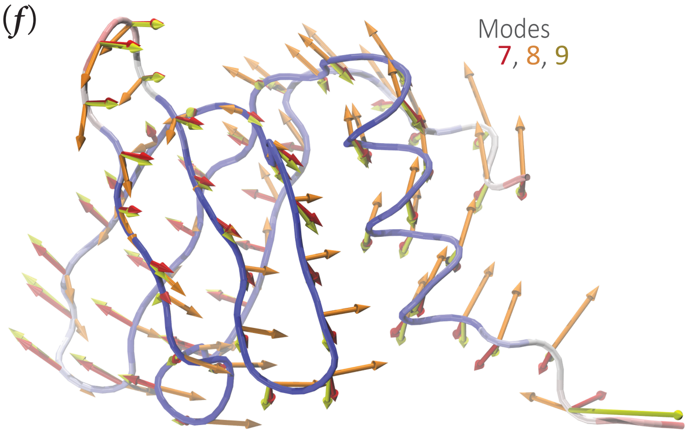

Figure S7: Difference electron density maps for the *Aae* Hfq•U_6 dataset solved in either *P*6 or *P*1

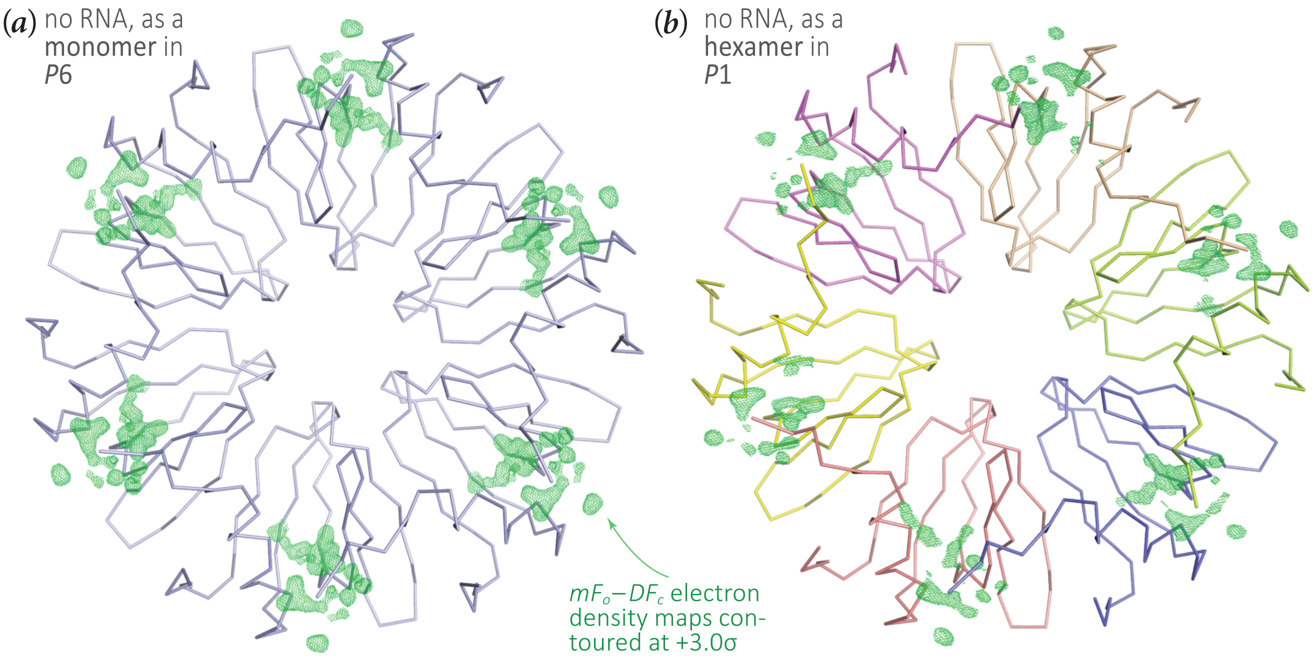

Figure S8: Chemical and geometric similarity of MPD to uridine, and fragments thereof: A case of small-molecule mimicry in the proximal RNA-binding site of Hfq?

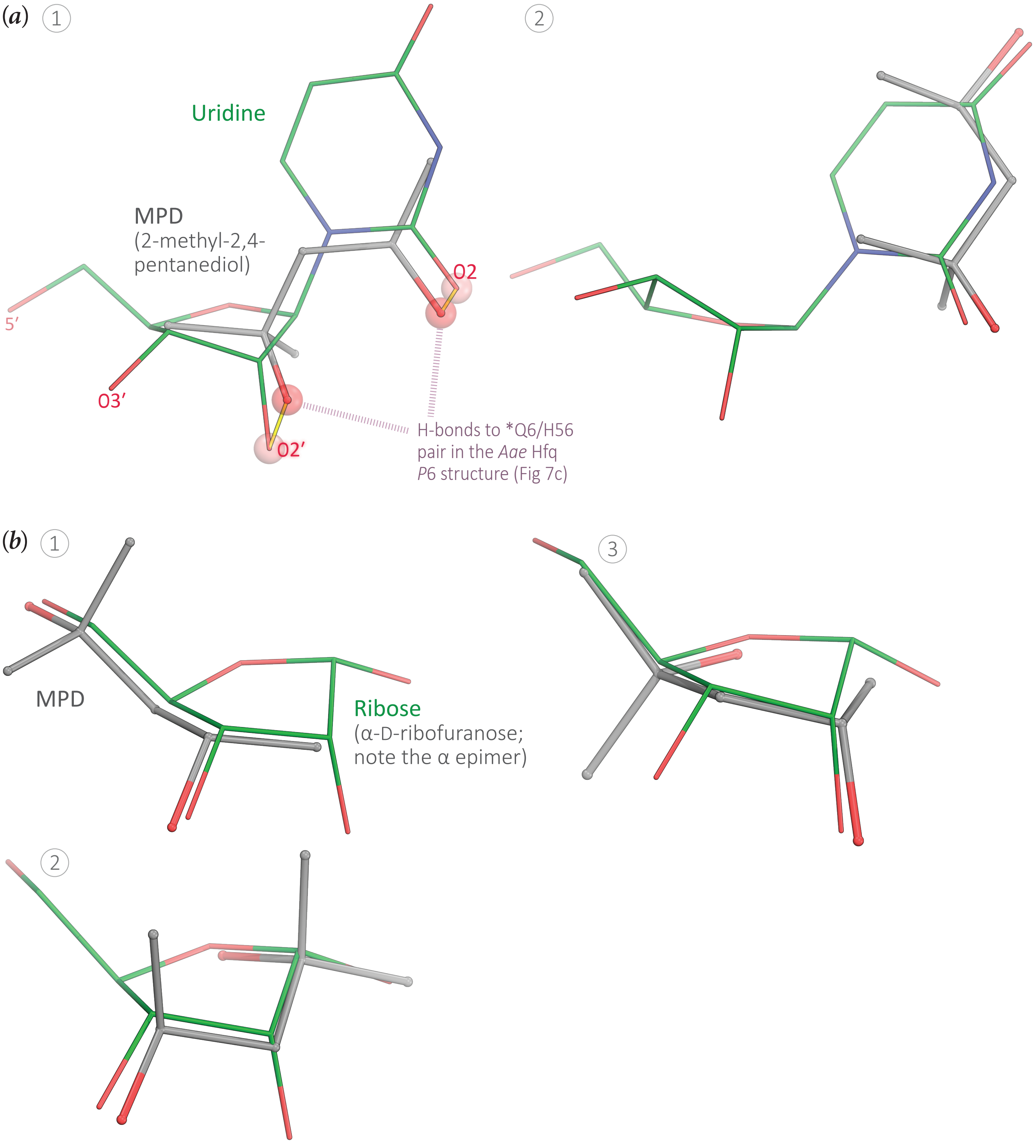

Figure S9: MPD can inhibit U-rich RNA binding to *Aae* Hfq

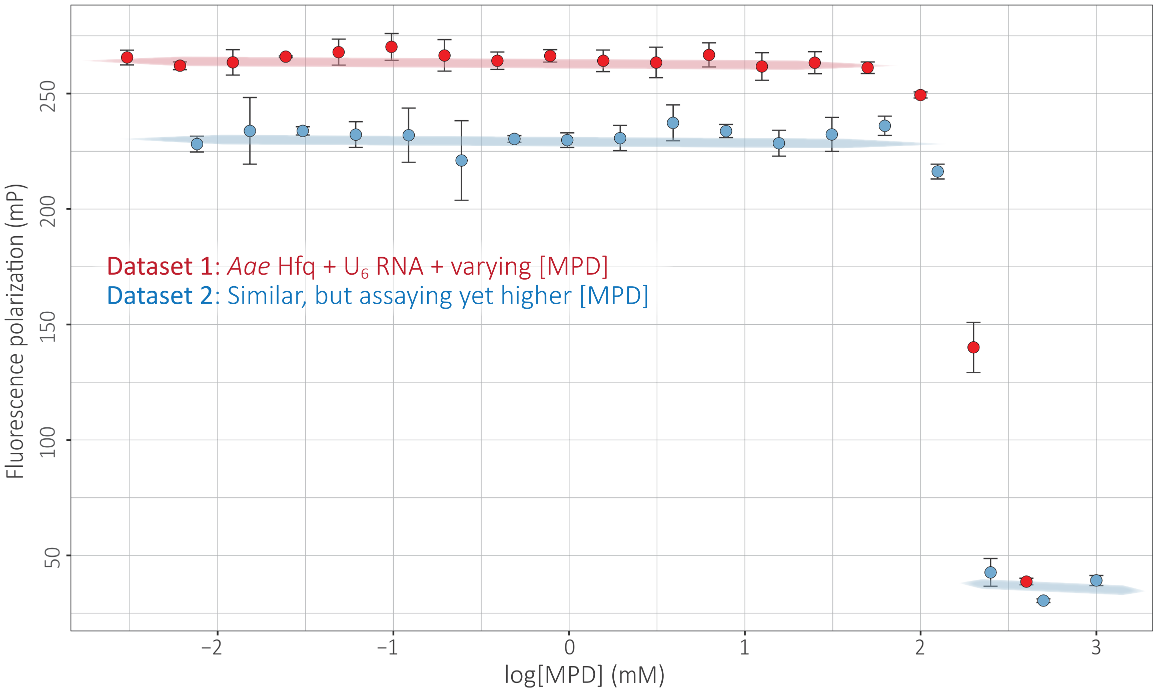

Supplement: Supplementary file 1 [file d-73-00294-sup1.pdf]
